# Investigating bile acid-mediated cholestatic drug-induced liver injury using a mechanistic model of multidrug resistance protein 3 (MDR3) inhibition

**DOI:** 10.3389/fphar.2022.1085621

**Published:** 2023-01-17

**Authors:** James J. Beaudoin, Kyunghee Yang, Jeffry Adiwidjaja, Guncha Taneja, Paul B. Watkins, Scott Q. Siler, Brett A. Howell, Jeffrey L. Woodhead

**Affiliations:** ^1^ DILIsym Services Division, Simulations Plus Inc., Research Triangle Park, NC, United States; ^2^ Division of Pharmacotherapy and Experimental Therapeutics, UNC Eshelman School of Pharmacy, University of North Carolina at Chapel Hill, Chapel Hill, NC, United States

**Keywords:** drug discovery, quantitative systems toxicology modeling, computational biology, bile duct, DILI (drug-induced liver injury), drug development

## Abstract

Inhibition of the canalicular phospholipid floppase multidrug resistance protein 3 (MDR3) has been implicated in cholestatic drug-induced liver injury (DILI), which is clinically characterized by disrupted bile flow and damage to the biliary epithelium. Reduction in phospholipid excretion, as a consequence of MDR3 inhibition, decreases the formation of mixed micelles consisting of bile acids and phospholipids in the bile duct, resulting in a surplus of free bile acids that can damage the bile duct epithelial cells, i.e., cholangiocytes. Cholangiocytes may compensate for biliary increases in bile acid monomers *via* the cholehepatic shunt pathway or bicarbonate secretion, thereby influencing viability or progression to toxicity. To address the unmet need to predict drug-induced bile duct injury in humans, DILIsym, a quantitative systems toxicology model of DILI, was extended by representing key features of the bile duct, cholangiocyte functionality, bile acid and phospholipid disposition, and cholestatic hepatotoxicity. A virtual, healthy representative subject and population (*n* = 285) were calibrated and validated utilizing a variety of clinical data. Sensitivity analyses were performed for 1) the cholehepatic shunt pathway, 2) biliary bicarbonate concentrations and 3) modes of MDR3 inhibition. Simulations showed that an increase in shunting may decrease the biliary bile acid burden, but raise the hepatocellular concentrations of bile acids. Elevating the biliary concentration of bicarbonate may decrease bile acid shunting, but increase bile flow rate. In contrast to competitive inhibition, simulations demonstrated that non-competitive and mixed inhibition of MDR3 had a profound impact on phospholipid efflux, elevations in the biliary bile acid-to-phospholipid ratio, cholangiocyte toxicity, and adaptation pathways. The model with its extended bile acid homeostasis representation was furthermore able to predict DILI liability for compounds with previously studied interactions with bile acid transport. The cholestatic liver injury submodel in DILIsym accounts for several processes pertinent to bile duct viability and toxicity and hence, is useful for predictions of MDR3 inhibition-mediated cholestatic DILI in humans.

## 1 Introduction

Drug-induced liver injury (DILI) is the most common cause of acute liver failure (Padda et al., 2011), and a major public health problem affecting patients, healthcare providers, drug developers and regulators (Mosedale and Watkins, 2017). Furthermore, the occurrence of DILI is an important reason for the suspension or early termination of drug development programs (Mosedale and Watkins, 2017). Understanding the mechanisms underlying different types of DILI (e.g., bile acid (BA)-mediated hepatocellular vs. bile duct injury) is critical to ongoing efforts to predict and ultimately prevent DILI events.

Disruption of BA homeostasis, particularly the hepatocellular accumulation of toxic BAs due to BA efflux inhibition, is a well-recognized mechanism of DILI. While reduced function of basolateral efflux transporters in the hepatocyte (e.g., multidrug resistance-associated protein (MRP) 3, MRP4, organic solute transporter (OST) α/β) is believed to contribute to this phenomenon, inhibition of the canalicular bile salt export pump (BSEP) is most often implicated (Morgan et al., 2013; Aleo et al., 2017; Beaudoin et al., 2020). The ensuing hepatocellular injury (e.g., apoptosis, necrosis) leads to the release of alanine and aspartate aminotransferases (ALT and AST) which are used clinically, among other biomarkers including total bilirubin (TB) alkaline phosphatase (ALP) and γ-glutamyl-transferase (GGT), to characterize the extent and origin of hepatotoxicity (i.e., hepatocellular, cholestatic or mixed). BSEP inhibition can lead to a decrease in bile flow (i.e., cholestasis) in the biliary tree that is BA-dependent, but the pattern of liver injury associated with reduced BSEP-mediated biliary excretion and the resultant accumulation of BAs often presents itself as hepatocellular (i.e., primarily involving hepatocytes as the injured cell type, which by convention is defined as ALT > 5x ULN, or when ALT/ALP (fold ULN) > 5 (Yang et al., 2013; Church and Watkins, 2021)). In contrast, clinically defined cholestatic liver injury (ALP > 2x ULN in combination with a major elevation of GGT and ALT/ALP (fold ULN) < 2 (Yang et al., 2013)) is characterized by injury to cholangiocytes and/or the canalicular membrane of hepatocytes (Mosedale and Watkins, 2020). More severe cholestasis is furthermore accompanied by elevations of conjugated bilirubin (Kullak-Ublick, 2013; Yang et al., 2013). While mixed liver injury (when ALT/ALP (fold ULN) is between 2 and 5) is the intermediate between the biochemically defined thresholds for hepatocellular and cholestatic liver injury, a mixed pattern is considered to have more in common with cholestatic than hepatocellular injury (Bjornsson and Jonasson, 2013). Based on several studies of DILI incidence, liver injury with a hepatocellular pattern is the most common (48%–58%), followed by cholestatic (20%–40%) and mixed (12%–22%) patterns (Bjornsson and Jonasson, 2013). In addition, mortality rates for DILI with hepatocellular, cholestatic and mixed patterns were shown to be 7%–18%, 5%–14% and 2%–5%, respectively (Bjornsson and Jonasson, 2013).

Drug-induced cholestasis is an issue of rising concern among drug developers and regulators (Mosedale and Watkins, 2020) and comes in multiple acute and chronic forms, involving injury ranging from impaired bile flow at the canalicular membrane of hepatocytes to obstruction of downstream portions of the bile duct (Padda et al., 2011). These multiple forms of drug-induced cholestasis include: 1) acute drug-induced cholestasis involving bile duct injury but minimal hepatocellular injury; 2) one of various chronic drug-induced cholangiopathies, such as a) non-specific bile duct injury with mild elevations of ALP or GGT, b) sclerosing cholangitis, c) mild reduction in the number of bile ducts (i.e., ductopenia), or d) progressive forms of the vanishing bile duct syndrome (VBDS); while 3) acute drug-induced cholestasis may occur without hepatitis, often with canalicular dilatation and bile plugs (pure or bland cholestasis); or 4) by portal inflammation and varying degrees hepatocellular injury (cholestatic hepatitis), the latter of which is the most common form of drug-induced cholestatic liver injury (Padda et al., 2011; Bjornsson and Jonasson, 2013; Yang et al., 2013). Some of these forms of drug-induced cholestasis mimic intrahepatic and extrahepatic cholestatic diseases, highlighting the importance of identifying the offending drug to avoid exacerbation of potential liver injury (Padda et al., 2011). Cholestatic DILI is usually mild and liver biochemistries eventually return to normal upon discontinuation of administering the drug, but in the worst case could lead to potentially irreversible VBDS and decompensated liver disease (Bjornsson and Jonasson, 2013). While bile duct loss in DILI is relatively uncommon, it has an estimated, overall mortality rate of 27%, and can lead to VBDS (Bonkovsky et al., 2017).

A key mechanism that is increasingly being proposed to underpin varying subtypes of cholestatic DILI is the inhibition of multidrug resistance protein 3 (MDR3), a phospholipid (PL) floppase residing on the canalicular membrane of hepatocytes along with BSEP and other canalicular transporters, including P-glycoprotein (P-gp)/MDR1, MRP2, ATP-binding cassette sub-family G (ABCG) 5 and 8, each with their own substrate selectivity (e.g., various drugs, primarily anionic compounds, cholesterol, respectively). Although MDR3 and MDR1 are highly homologous, MDR3 does not confer drug resistance, and instead preferentially mediates the translocation of the PL subtype phosphatidylcholine (PC) from the inner to the outer leaflet of the canalicular membrane, which thereby becomes available for biliary excretion (Boyer, 2013). Other PL subtypes as part of the canalicular membrane include sphingomyelin, phosphatidylethanolamine, and phosphatidylserine, but PC is the primary PL found in bile (Boyer, 2013). BAs that have been excreted into the bile canaliculus by BSEP stimulate biliary PC excretion by extracting this PL subtype from the luminal side of the canalicular membrane (Boyer, 2013). BAs and PLs form mixed micelles with cholesterol, thereby greatly reducing the toxicity associated with lipophilic, free biliary BA monomers. Disruption of MDR3 activity results in a reduced availability of PC in the bile canaliculus, leading to impaired biliary formation of mixed micelles, and predisposing cholangiocytes in the biliary epithelium to BA-mediated injury or the generation of cholesterol crystals (Morita and Terada, 2014; Mosedale and Watkins, 2017).

The clinical relevance of MDR3 is also highlighted by phenotypes associated with mutations in *ABCB4*, the gene that encodes the PC floppase. Specific variants in *ABCB4* result in impairment of MDR3-mediated PC translocation and lead to a spectrum of cholestatic disorders of varying severity. Progressive familial cholestasis (PFIC) type 3, characterized by homozygous MDR3 loss-of-function mutations, is an aggressive form of inherited cholestasis. Other variants or heterozygosity in *ABCB4* can lead to PL-associated cholelithiasis (gallstone disease) or intrahepatic cholestasis of pregnancy (ICP) in women, while *ABCB4* variants also associate with elevated liver function tests, gallbladder and bile duct carcinoma and liver cirrhosis (Boyer, 2013; Andress et al., 2014; Stättermayer et al., 2020). It is unclear whether other acquired human cholestatic liver diseases such as primary sclerosing cholangitis (PSC) or primary biliary cholangitis (PBC) are linked to *ABCB4* mutations, but mice with Abcb4 impairment do spontaneously develop sclerosing cholangitis with features resembling human PSC (Trauner et al., 2008; Boyer, 2013). Histologically, PFIC3 is characterized by portal inflammation, fibrosis, progression to cirrhosis, and unlike PFIC2 (another type of progressive familial cholestasis characterized by mutations in the *ABCB11* gene encoding BSEP), bile ducts are highly proliferative in PFIC3 (Oude Elferink and Paulusma, 2007). While biliary PL levels are low and GGT levels are high in PFIC3, this is not the case for PFIC2 (Boyer, 2013).

Furthermore, polymorphisms and rare mutations in MDR3, as well as BSEP, have been associated with an increased risk for DILI from compounds such as oral contraceptives, psychotropic drugs, proton pump inhibitors, and various antibiotics (Bjornsson and Jonasson, 2013), indicating that functional impairment of these proteins predisposes to hepatotoxicity (Lang et al., 2007). In contrast to drug-mediated BSEP inhibition, which has been widely investigated, interactions of drugs with MDR3 are understudied, and currently our understanding of the role of MDR3 in cholestatic DILI pathogenesis is relatively limited (Mahdi et al., 2016; Aleo et al., 2017). The hepatotoxic drugs itraconazole and chlorpromazine have been shown to inhibit MDR3 activity while only weakly inhibiting BSEP, indicating that MDR3 dysfunction may play a more important role than BSEP inhibition in the pathogenesis of DILI for these compounds (Mosedale and Watkins, 2017). In the case of itraconazole administration, ALP, GGT and TB were elevated in multiple patients, suggesting drug-induced cholestasis liability, but based on some elevations in ALT and AST, this drug may also lead to hepatocellular injury to a certain degree (Yoshikado et al., 2011). In other cases, such as for the DILI-associated compounds ketoconazole, benziodarone, ritonavir and tipranavir, substantial inhibition of both MDR3 and BSEP seem to be at play, suggesting that simultaneous inhibition of these two canalicular proteins could confer DILI susceptibility as well, and potentially aggravate liver injury (Mahdi et al., 2016; Aleo et al., 2017; Mosedale and Watkins, 2017).

In the current work, a quantitative systems toxicology (QST) model of drug-induced hepatotoxicity, DILIsym, was extended to mechanistically represent components putatively contributing to clinically defined cholestatic liver injury, and to allow for the assessment of toxicity induced by MDR3 inhibition. Cholangiocellular handling of, and responses to biliary BAs have been incorporated as potential adaptation mechanisms to biliary BA toxicity. The construction of this new submodel, as detailed in subsequent sections of this paper, is the latest addition to this computational modeling platform, with the goal to further improve our understanding and predictions of DILI in humans, which can enhance patient safety, diminish the need for animal testing, and decrease the resources required for new drug development. The effort behind the continuous DILIsym development since 2012 has been supported by the DILI-sim Initiative, a pre-competitive partnership between DILIsym Services Division, Simulations Plus Inc. and multiple pharmaceutical companies (Howell et al., 2016). The modeling platform combines drug and metabolite exposure at the site of interaction with hepatotoxicity mechanisms to delineate the impact of these compounds, at specific concentrations, on the cellular, organ and whole organism levels (Mosedale and Watkins, 2017). Various prospective and retrospective applications of DILIsym have provided support that DILI liability of a compound can be attributed to three key mechanisms: mitochondrial dysfunction, oxidative stress, and alterations in BA homeostasis (Howell et al., 2012; Shoda et al., 2014; Longo et al., 2016; Mosedale and Watkins, 2017; Woodhead et al., 2018; 2020; 2022; Generaux et al., 2019). The present work describes the new cholestatic liver injury-related components that were incorporated into the model, which extends the original BA homeostasis model, and has the potential to improve the predictive capability of the DILIsym model as a whole.

## 2 Materials and methods

**FIGURE 1:**
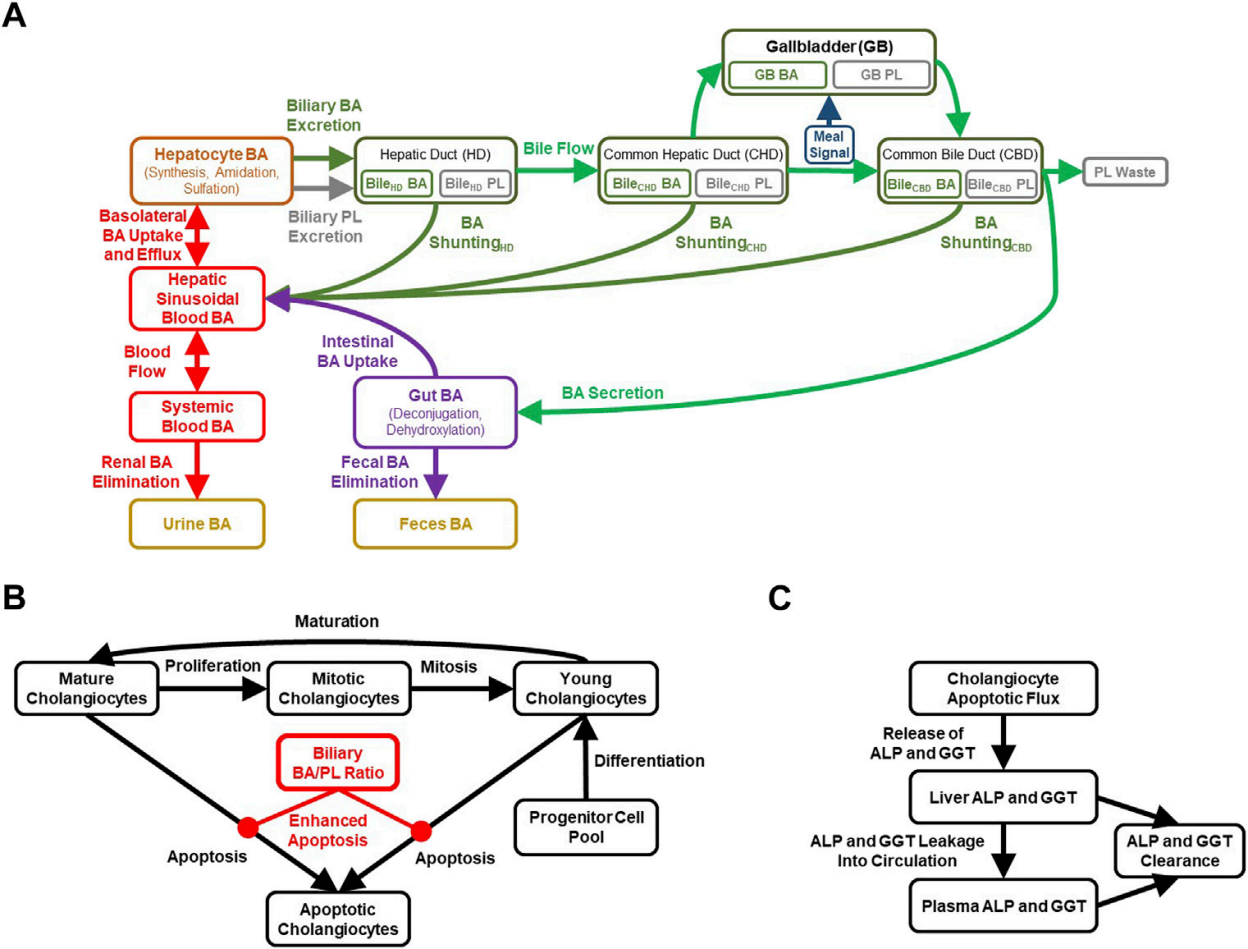
Diagrammatic representation of the cholestatic liver injury model, including features related to **(A)** BA and PL homeostasis, **(B)** cholangiocyte life cycle for each of the three bile duct segments, **(C)** cholestatic liver injury biomarkers. ALP, alkaline phosphatase; BA, bile acid; GGT, gamma-glutamyl-transferase; PL, phospholipid.

DILIsym (version 8A), a commercially available QST software platform, was used as a starting place for the development of a mechanistic submodel of MDR3 inhibition-mediated cholestatic DILI using MATLAB R2021a. The model consists of previously constructed and validated representations of BA homeostasis (Woodhead et al., 2014b; 2014a), mitochondrial function (Yang Y. et al., 2014), oxidative stress (Howell et al., 2012; Howell et al., 2014; Woodhead et al., 2012), innate immunity (Shoda et al., 2017), the hepatocyte life cycle, clinical biomarkers, drug metabolism and distribution, which are combined and mathematically solved in DILIsym. In the present work, the existing BA homeostasis model was expanded to represent bile flow-mediated BA movement along the bile duct and to include a cholehepatic shunt pathway for BAs (Figure 1A). Furthermore, representations of biliary PL excretion, different modes of MDR3 inhibition, the cholangiocyte life cycle, biliary BA toxicity, as well as biliary bicarbonate (HCO_3_^−^) secretion and its effects on cholehepatic shunting, bile flow and BA toxicity have been developed, as described in more detail below. The new submodel is being added to the next commercially available version of DILIsym.

### 2.1 Bile acid homeostasis

BA disposition is a critical component of the newly developed cholestatic liver injury submodel, mainly because the movement of variable quantities of BAs along the biliary tree is an important determinant of BA-mediated cholangiocyte injury. Extension of the BA homeostasis model in the current work required recalibration and revalidation of the overall BA disposition in the model. Key considerations of the BA homeostasis model in DILIsym (Figure 1A) are summarized in the Supplementary Material.

### 2.2 Bile formation

The production of bile is unique to the liver and serves an important physiological function by providing an excretory route for endogenous and exogenous compounds, and to solubilize lipids in the intestinal lumen (Boyer, 2013; Arab et al., 2017). Bile is produced both by hepatocytes and cholangiocytes, and is comprised of water (∼95%), various endogenous compounds including BAs, PLs, HCO_3_^−^, cholesterol, bilirubin, glutathione, porphyrins, vitamins, and exogenous compounds such as certain drugs that undergo biliary excretion (Boyer, 2013; Arab et al., 2017). Bile formation is an osmotic process that attracts water *via* aquaporins and through intercellular tight junctions, and is driven by the concentration gradient produced by hepatocyte- and cholangiocyte-mediated active transport processes (Boyer, 2013). DILIsym represents biliary excretion of BAs *via* BSEP, which is a major driving force of bile flow. Biliary BAs rapidly become incorporated into mixed micelles consisting of PLs and cholesterol, thereby greatly diminishing the toxic potential of free BAs in the bile duct lumen (Boyer, 2013).

The biliary BA/PL ratio can be used to represent mixed micelle formation, and in the case of a high ratio, reflects a toxic surplus of free BA monomers in the bile. The BA/PL ratio has been associated with cholestatic liver injury in several studies (Geuken et al., 2004; Buis et al., 2009; Davit-Spraul et al., 2009), which is described in more detail in the Supplementary Material. The DILIsym model has been parameterized to enhance cholangiocyte apoptosis when the biliary BA/PL ratio exceeds a value of 10, with higher ratios inducing more toxicity and thus cholangiocyte apoptosis (Figure 1B). This parameterization was based mainly on the relationship between biliary BA/PL and bile duct injury reported in liver transplant patients (Geuken et al., 2004). Furthermore, a time-dependent relationship between biliary BA/PL ratios and the release of cholestatic liver injury biomarkers (ALP, GGT) was reported (Geuken et al., 2004), and incorporated into the model using a simplified representation involving cholangiocyte injury as the sole contribution to the release of these biomarkers (Figure 1C). Finally, MDR3, similar to BA transporters, is regulated by BA-activated farnesoid X receptor (FXR) (Huang et al., 2003; Boyer, 2013) and has been represented in the model, which could help with maintaining the biliary BA/PL ratio at relatively constant levels.

Concentrations of PC (13.3 ± 1.76 nmol/mg tissue) have been reported in human liver (*n* = 8) (Kotronen et al., 2010), and are assumed to be available for biliary excretion within the model. MDR3 plays a critical role in the translocation of PC on the canalicular membrane to allow for the biliary formation of mixed micelles containing these PLs. However, some literature reports suggest that even in the absence of MDR3, a fraction of PC may still be available for biliary excretion *via* an MDR3-independent mechanism (Davit-Spraul et al., 2009; Yoshikado et al., 2011); in the model, 50% of PC is excreted into bile in an MDR3-independent fashion. MDR3-mediated PC translocation has been found to follow Michaelis-Menten kinetics, with a Michaelis-Menten constant (K_M_) of 16.6 ± 2.6 µM (Kluth et al., 2015). Furthermore, a cryogenic electron microscopy structure of MDR3 rationalizes why MDR3 has specificity for PC relative to other PLs based on protein-substrate interactions that can stabilize the choline moiety of PC (Nosol et al., 2021). The molecular structure also provides insights into substrate- and inhibitor-binding sites of MDR3, by showing that the MDR3 inhibitor posaconazole can block PLs from reaching the central cavity as a potential mechanism of floppase activity inhibition (Nosol et al., 2021). This motivated the inclusion and exploration of multiple modes of MDR3 inhibition in the newly developed DILIsym model utilizing the general mixed inhibition equation that describes different modes of inhibition based on an α parameter: competitive (very large α), non-competitive (α = 1) and mixed inhibition (α between the special cases of competitive and non-competitive inhibition), similar to the modes of hepatocellular BA transport inhibition that are available in the model.

Equations for competitive, mixed, and non-competitive inhibition types are as follows:

$$\begin{array}{c} Competitive:\;V=\frac{V_{\max}\times S}{K_{M}\times \left(1+\frac{I}{K_{i}}\right)+S}\\ Mixed:\;V=\frac{V_{\max}\times S}{K_{M}\times \left(1+\frac{I}{K_{i}}\right)+S\times \left(1+\frac{I}{{\alpha \times K}_{i}}\right)}\\ Non-Competitive:\;V=\frac{V_{\max}\times S}{K_{M}\times \left(1+\frac{I}{K_{i}}\right)+S\times \left(1+\frac{I}{K_{i}}\right)} \end{array}$$

where V represents velocity (e.g., PL efflux), V_max_ is the maximum velocity without inhibitor, S is the substrate concentration, K_M_ is the Michaelis-Menten constant, I is the inhibitor concentration, K_i_ is the inhibition constant, and the α constant determines the extent to which binding of the inhibitor alters the affinity of the protein for the substrate.

### 2.3 Intrahepatic and extrahepatic bile ducts

While the gallbladder and its role in BA storage and meal effects have been represented in DILIsym previously, three key segments of the bile duct have been incorporated in the new version of the model in downstream order: the intrahepatically located hepatic duct (HD), and the extrahepatically located common hepatic duct (CHD) and common bile duct (CBD). In the model, the HD represents the entire intrahepatic biliary drainage system, including the bile canaliculi, canals of Hering, ductules, interlobular ducts, septal ducts, and the increasingly larger intrahepatic ducts (Boyer, 2013; Ramesh Babu and Sharma, 2014). The intrahepatic ducts converge to form the CHD extrahepatically (Nakanuma et al., 1997; Ramesh Babu and Sharma, 2014; Higashiyama et al., 2016). The cystic duct branches off from the CHD and carries bile to the gallbladder. The cystic duct is not explicitly represented in the model; instead, the majority of bile from the CHD flows directly to the gallbladder. The CBD is formed further downstream of the CHD and primarily receives bile that was stored in the gallbladder, but also a portion of bile that was restricted to the CHD. The CBD extends downward to reach the duodenum into which the CBD releases its biliary contents. Differences between the basal secretion of hepatic bile and the rate of hepatic bile entering the gallbladder, in addition to the fraction of the BA pool that accumulates in the gallbladder suggest that ∼20% of bile flow bypasses the gallbladder (Turumin et al., 2013), which was incorporated into the model. To estimate the luminal volumes of each of the bile duct segments, literature data on HD, CHD and CBD volumes, diameters, surface areas and lengths were leveraged (Bruneton et al., 1981; Ludwig et al., 1998; Gray et al., 2005; Hoeffel et al., 2006; Blidaru et al., 2010; Boyer, 2013; Ramesh Babu and Sharma, 2014; Gómez Zuleta et al., 2017). Furthermore, these data, along with total cholangiocyte number estimates (Banales et al., 2019), were used to inform approximate numbers of cholangiocytes in the various bile duct segments.

### 2.4 Cholangiocytes

Cholangiocytes are the epithelial cells lining the intrahepatic and extrahepatic bile ducts, and are highly specialized cells contributing to the production and homeostasis of bile (Baiocchi et al., 2019; Banales et al., 2019). Although it is increasingly appreciated that cholangiocellular size, structure and function vary based on their location along the bile duct (Boyer, 2013; Baiocchi et al., 2019; Banales et al., 2019; Brevini et al., 2020), the newly constructed representation of cholangiocytes in DILIsym currently assumes similar properties for cholangiocytes in the HD, CHD, and CBD. Cell differentiation, proliferation and secretion is regulated by primary cilia located on the apical membrane of all cholangiocytes (Boyer, 2013). A critical function of cholangiocytes includes the fluidization and alkalinization of canalicular bile, which involves multiple cholangiocellular uptake and efflux transport processes, ultimately resulting in the secretion of an HCO_3_^−^-rich fluid (Boyer, 2013). Approximately 30%–40% of daily bile production in humans is attributed to cholangiocytes, and constitutes an important part of BA-independent bile flow. As cholangiocytes are exposed to high, millimolar concentrations of biliary BAs with detergent-like properties, the secretory functions of the biliary epithelium participate in protecting cholangiocytes against BA-mediated toxicity.

The model includes a simplified cholangiocyte life cycle (Figure 1B) for each of the three bile duct segments, representing young, mature and mitotic cholangiocytes, as well as a maturation rate (young cholangiocytes → mature cholangiocytes), proliferation rate (mature cholangiocytes → mitotic cholangiocytes), and mitotic rate (mitotic cholangiocytes → young cholangiocytes). Cholangiocytes undergo apoptosis, and this pathway is sensitized in certain cholangiopathies such as PBC, in which HCO_3_^−^ secretion is reduced (Hohenester et al., 2012; Chang et al., 2016); thus, basal apoptosis from either the mature or young cholangiocyte pool is represented in the model. Furthermore, cholangiocytes are highly proliferative cells allowing for regeneration of the biliary epithelium in response to acute injury (Brevini et al., 2020). Nevertheless, regeneration of cholangiocytes is thought to be slower than that of hepatocytes, in part because cholestatic DILI reactions are inclined to linger after cessation of the offending drug, although immune responses may play a role in that protracted recovery as well (Padda et al., 2011). To accommodate regeneration of the bile duct epithelium, the model includes a differentiation rate allowing for the generation of new, young cholangiocytes. Biliary BA-mediated toxicity, represented by a biliary BA/PL ratio >10, is included in the model to enhance the apoptotic rate in the cholangiocyte life cycle (and thereby releasing ALP and GGT; Figure 1C), effectively diminishing the total pool of viable cholangiocytes.

### 2.5 Cholehepatic shunting of bile acids

While the enterohepatic circulation (EHC) of BAs is a well-recognized component of BA homeostasis, the cholehepatic shunt, involving cholangiocytes instead of intestinal cells to cycle BAs, is a relatively understudied and poorly understood alternative pathway for BAs (see Supplementary Material for more details). Nevertheless, the cholehepatic shunt is hypothesized to play an important physiological role, particularly in the context of cholestatic conditions (e.g., in the case of extrahepatic obstruction) when cholangiocytes may be exposed to excessive concentrations of free, biliary BAs (Xia et al., 2006).

Based on literature supporting a role for the existence of the cholehepatic shunt pathway, a representation of this pathway for BAs has been constructed for each of the three bile duct segments in DILIsym: HD, CHD and CBD (Figure 1A). For simplicity and due to data limitations, it is currently assumed in the model that the rate of shunting, involving a lumped parameter representing both passive diffusion and transporter-mediated processes, is similar for all BA species. The model assumes that the rate of cholehepatic shunting is most substantial in the intrahepatic HD, followed by the more downstream CHD and CBD segments of the extrahepatic bile duct. Furthermore, the model assumes that the rate of transport along the shunt pathway is only a fraction of the rate of bile flow (e.g., the rate of BA shunting in the HD has arbitrarily been set to ∼2% of the total rate of bile flow-driven BA transport along the bile duct—but still allowing for the exploration of both enhancement and inhibition of this BA pathway). The cholehepatic shunt pathway representation in the model allows for being impacted by HCO_3_^−^ secretion, as will be described in more detail in the next section.

### 2.6 Bicarbonate secretion

Under normal conditions, cholangiocytes are continuously exposed extracellularly to millimolar concentrations of biliary BAs, whereas other cell types including hepatocytes are susceptible to BA-induced toxicity at micromolar concentrations (Hohenester et al., 2011). Anion exchanger 2 (AE2)-mediated secretion of HCO_3_^−^ (in exchange for a chloride anion) by cholangiocytes is hypothesized to protect the biliary epithelium against high concentrations of toxic, detergent-like BAs passing through the bile duct. AE2 protein was found to be expressed on the apical membrane of cholangiocytes in small and medium bile ducts (Boyer, 2013). Biliary HCO_3_^−^ secretion alkalinizes the bile, thereby increasing the pH of bile, and altering the ionization status of various biliary BAs. Data suggest that BA protonation influences transporter-independent penetration of BAs into cholangiocytes (Hohenester et al., 2012), and the protonation of certain BAs may be particularly impacted by mild changes in the physiological pH range. For example, according to the Henderson-Hasselbalch equation, unconjugated chenodeoxycholic acid (CDCA) and its glycine-conjugated form (GCDCA) have a pKa around 4.5 and 4.2, respectively, and their protonation is highly sensitive to changes in pH around the physiologic range (Hohenester et al., 2011). *In vitro* experiments demonstrated pH dependence for cholangiocellular uptake of GCDCA, and for cytotoxicity induced by CDCA and GCDCA (Hohenester et al., 2011; 2012). More specifically, when extracellular pH was experimentally reduced from 7.4 to 6.4, uptake of GCDCA more than tripled, and apoptosis-associated caspase levels increased ∼10- and ∼30-fold when incubated with 0.5 mM CDCA or 1 mM GCDCA, respectively (Hohenester et al., 2011; Hohenester et al., 2012). In addition to experimentally decreasing extracellular pH, knocking down AE2 in immortalized human cholangiocytes also resulted in increased BA uptake and toxicity (Hohenester et al., 2012). Additional details are provided in the Supplementary Material.

The model represents HCO_3_^−^ secretion in the HD, CHD and CBD, and utilizes a relationship reported between biliary HCO_3_^−^ and pH for human donor livers (Matton et al., 2019). Livers with a low bile duct injury score were associated with higher biliary HCO_3_^−^ concentrations and biliary pH compared to livers with a high bile duct injury score, providing support for the protective function of bile alkalization (Matton et al., 2019). Based on the observed effects of biliary HCO_3_^−^ on the cholangiocellular penetration of CDCA and GCDCA and BA-induced toxicity (Hohenester et al., 2012), the model has been parameterized in a manner that results in reduced cholehepatic shunting and BA toxicity when biliary HCO_3_^−^ secretion increases. The increase in biliary HCO_3_^−^ furthermore leads to enhanced biliary water secretion through the effects of osmosis resulting in higher bile flow, which has also been represented in the model. Biliary HCO_3_^−^ concentrations are represented at baseline, and has been parameterized in a manner to increase in response to elevations in the biliary BA/PL ratio, reflecting the stimulation of BA-dependent TGR5 signaling in a biliary BA concentration-dependent manner.

### 2.7 Model calibration and validation

Publicly available data were used to calibrate and validate the newly developed cholestatic liver injury submodel in DILIsym (see Results section). Model parameters related to BA and PL homeostasis and bile duct physiology were calibrated for a representative, healthy adult human of 70 kg using a variety of literature data informing model inputs (e.g., BA synthesis) and outputs (e.g., biliary BA/PL ratio). This approach was extended to generate the Human_ROS_apop_mito_BA_chol_v1 SimPops^®^, a virtual population of 285 subjects with variability in parameters relevant to evaluate predictions of DILI caused by drugs through RNS-ROS generation, direct mitochondrial function disruption, BA transport inhibition and/or MDR3 inhibition. While parameters related to oxidative stress and mitochondrial impairment were kept intact from the earlier version of this SimPops (*n* = 285), all BA parameters were updated. Variability around input parameters as well as model outputs were supported with literature data in many cases, while in other cases assumptions were made mainly due to data limitations or knowledge gaps (Table 1). To validate the SimPops, simulations were performed without drug treatment (i.e., SimPops baseline simulations), and separately with one of several drug treatment protocols serving as positive or negative controls.

**TABLE 1:** Bile Acid and Phospholipid Toxicity Mechanism Parameters with Distribution of Values for Virtual Subjects in the Human_ROS_apop_mito_BA_chol_v1 SimPops. ASBT, apical sodium–BA transporter; BA, bile acid; BSEP, bile salt export pump; CDCA, chenodeoxycholic acid; LCA, lithocholic acid; MDR3, multidrug resistance protein 3; PL, phospholipid; Vmax, maximum reaction velocity.

SimPops parameter category	Parameter symbol	Data source for distribution
Hepatic BA uptake transporter Vmax values	BA_uptake_Vmax	All BA transporters were assumed to have the same distribution as human BSEP reported in Meier et al. (2006); similar expression ranges are also reported in Bernhardt et al. (2012); all BA uptake transport Vmax values treated as covariates
	CDCA_uptake_Vmax	
	CDCAamide_uptake_Vmax	
	LCA_uptake_Vmax	
	LCAamide_uptake_Vmax	
	LCAsulfate_uptake_Vmax	
Hepatic basolateral BA efflux transporter Vmax values	BA_baso_Vmax	All BA transporters were assumed to have the same distribution as human BSEP reported in Meier et al. (2006); similar expression ranges are also reported in Bernhardt et al. (2012); all basolateral BA efflux Vmax values treated as covariates
	CDCA_baso_Vmax	
	CDCAamide_baso_Vmax	
	LCA_baso_Vmax	
	LCAamide_baso_Vmax	
	LCAsulfate_baso_Vmax	
Hepatic biliary BA and PL excretion transporter Vmax values	BA_canal_Vmax	All BA transporters were assumed to have the same distribution as human BSEP reported in Meier et al. (2006); similar expression ranges are also reported in Bernhardt et al. (2012); all biliary BA and PL excretion Vmax values treated as covariates
	CDCA_canal_Vmax	
	CDCAamide_canal_Vmax	
	LCA_canal_Vmax	
	LCAamide_canal_Vmax	
	LCAsulfate_canal_Vmax	
	Vmax_PL_canal	
Hepatic BA synthesis	BA_synthesis_So	Range and distribution based on data in Mok et al. (1977), Vantrappen et al. (1981)
	CDCA_synthesis_So	
Gut BA uptake	Vmax_BA_gut	Range and distribution based on report in Ho et al. (2011); all intestinal BA uptake Vmax values treated as covariates
	Vmax_CDCA_gut	
	Vmax_CDCAamide_gut	
	Vmax_LCA_gut	
	Vmax_LCAamide_gut	
	Vmax_LCAsulfate_gut	
Fecal BA elimination	k_out_bulk_BA	Assumed parameter range of ±2 orders of magnitude with ±50% standard deviation and validated with outcome data
	k_out_CDCA	
	k_out_CDCAamide	
	k_out_LCA	
	k_out_LCAamide	
	k_out_LCAsulfate	
Cholehepatic BA shunting	shunt_HD_o	Assumed to have same distribution as intestinal ASBT reported in Ho et al. (2011)
	shunt_CHD_o	
	shunt_CBD_o	
BA and PL regulation	uptake_reg_scale	Assumed parameter range of 0–8 with ±50% standard deviation and validated with outcome data; canal_PL_reg_scale (MDR3) and canal_reg_scale (BSEP) treated as covariates
	baso_reg_scale	
	canal_reg_scale	
	canal_PL_reg_scale	
Hepatic BA conjugation	CDCA_amidation_Vmax	Used 50-fold lognormal range of variability in metabolizing enzymes in line with variability reported in Tracy et al. (2016)
	LCAamide_sulfation_Vmax	
Gut BA metabolism	CDCA_deconjug_Vmax	Assumed parameter range of ±2 orders of magnitude with ±50% standard deviation and validated with outcome data
	LCA_synthesis_Vmax	
Renal BA elimination	Vmax_renal_BA	Assumed to have same distribution as BSEP reported in Meier et al. (2006)

### 2.8 Sensitivity analyses

The representative, healthy virtual subject was used in sensitivity analyses interrogating cholehepatic BA shunting and biliary HCO_3_^−^ secretion, two mechanisms hypothesized to be protective to the bile duct epithelium during cholestatic stress. The SimPops was used to assess the role of different modes of MDR3 inhibition (competitive, non-competitive, mixed) on BA and PL homeostasis, bile duct injury and adaptation mechanisms.

## 3 Results

**FIGURE 2:**
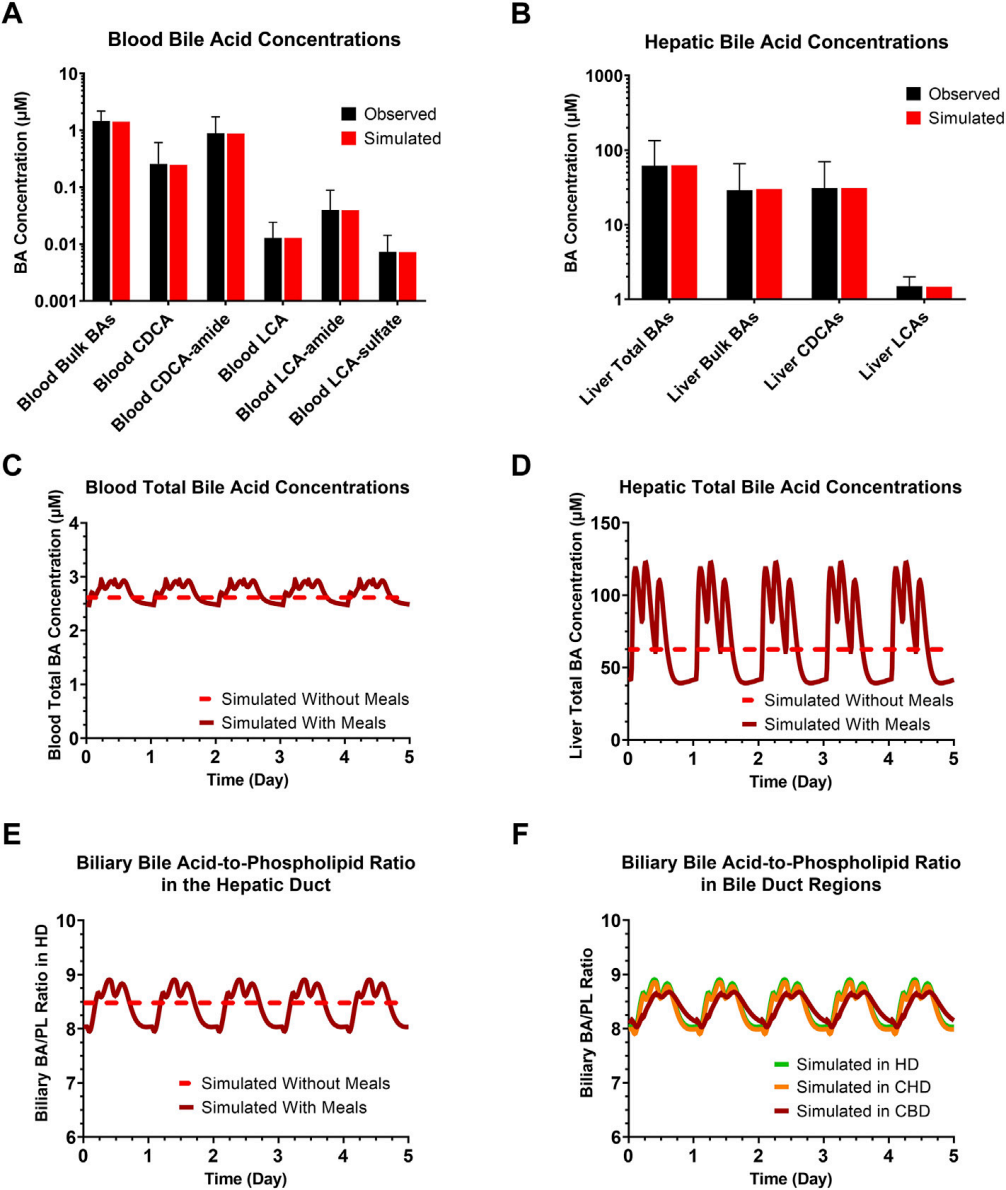
Clinically observed and simulated **(A)** blood BA concentrations and **(B)** liver BA concentrations. Observed data are presented as mean ± SD. Simulations with or without meals of blood total BA concentrations **(C)**, hepatic total BA concentrations **(D)**, the biliary BA/PL ratio in the HD **(E)**, and biliary BA/PL ratio in the HD, CHD and CBD **(F)**. BA, bile acid; CBD, common bile duct; CDCA, chenodeoxycholic acid; CHD, common hepatic duct; HD, hepatic duct; LCA, lithocholic acid; PL, phospholipid.

An extended submodel of BA and PL homeostasis, and representations of the cholangiocyte life cycle and cholestatic liver injury biomarkers were developed in DILIsym, with key features depicted in Figure 1. Calibration of the model for a representative, healthy virtual subject under fasted and fed conditions resulted in the recapitulation of a variety of published, clinical data. The simulated/observed ratios (%) for the blood concentrations of the six major BA categories were between 96.6%–100% (Trottier et al., 2011) (Figure 2A). Simulated/observed ratios (%) for hepatic BA concentrations were between 97.8%–103.1% (Setchell et al., 1997) (Figure 2B). In both blood and liver, the CDCA species and bulk BA species represented the large majority of all measured BAs; lithocholic acid (LCA) species, although the most hepatotoxic, only represented a minor fraction in these two matrices, consistent with the literature. Furthermore, the model reasonably recapitulated: 1) total BA pool (simulated: ∼2.6 g, observed: 1.2–6.6 g), 2) BA synthesis rate (simulated: ∼440 mg/day, observed: 175–1,250 mg/day), 3) BA pool that is lost (and replaced) on a daily basis (simulated: ∼16%, observed: ∼5–35%), 4) large majority of BA pool residing in the gut (simulated: ∼78%, observed: ∼90%), and 5) a small fraction of lost BAs being eliminated *via* urine (simulated: ∼0.6%, observed: <1%) (Subbiah et al., 1976; Mok et al., 1977; Vantrappen et al., 1981; Kullak-Ublick et al., 1995; Hofmann, 1999; Meier and Stieger, 2002). Additionally, the large majority of biliary BAs consists of CDCA species (simulated: ∼41%, observed: ∼35%) while only a minority consists of LCA species (simulated: ∼1%, observed: ∼1%); in contrast, due to gut bacteria-mediated BA metabolism, the large majority of fecal BAs consists of LCA species (simulated: ∼48%, observed: 32%), whereas only a minority consists of CDCA species (simulated: 2%, observed: 2%) (Baars et al., 2015), which is reasonably captured by the model. The model is capable of performing simulations under fasted and fed conditions (three meals per day); meal signals result in the contraction of the gallbladder, resulting in oscillatory BA concentrations in the blood and liver (Figures 2C, D), in agreement with reported, post-prandial increases in circulating BAs (LaRusso et al., 1974; Napolitano et al., 2014). Meals are also an important consideration for the biliary BA/PL ratio (Figure 2E), since oscillations in this ratio are to be expected with meals, and the model, based on its current parameterization, starts eliciting a cholestatic hepatotoxic response when this ratio reaches above the threshold value of 10 (Geuken et al., 2004). A healthy, representative individual is not expected to observe cholestatic liver injury under fasted nor fed conditions without drug treatment. Finally, based on the structural organization of individual bile duct segments, the occurrence of cholehepatic shunting of BAs, bile flow and gallbladder storage of BAs, subtle differences in the biliary BA/PL ratio were seen depending on the bile duct segment (Figure 2F).

**FIGURE 3:**
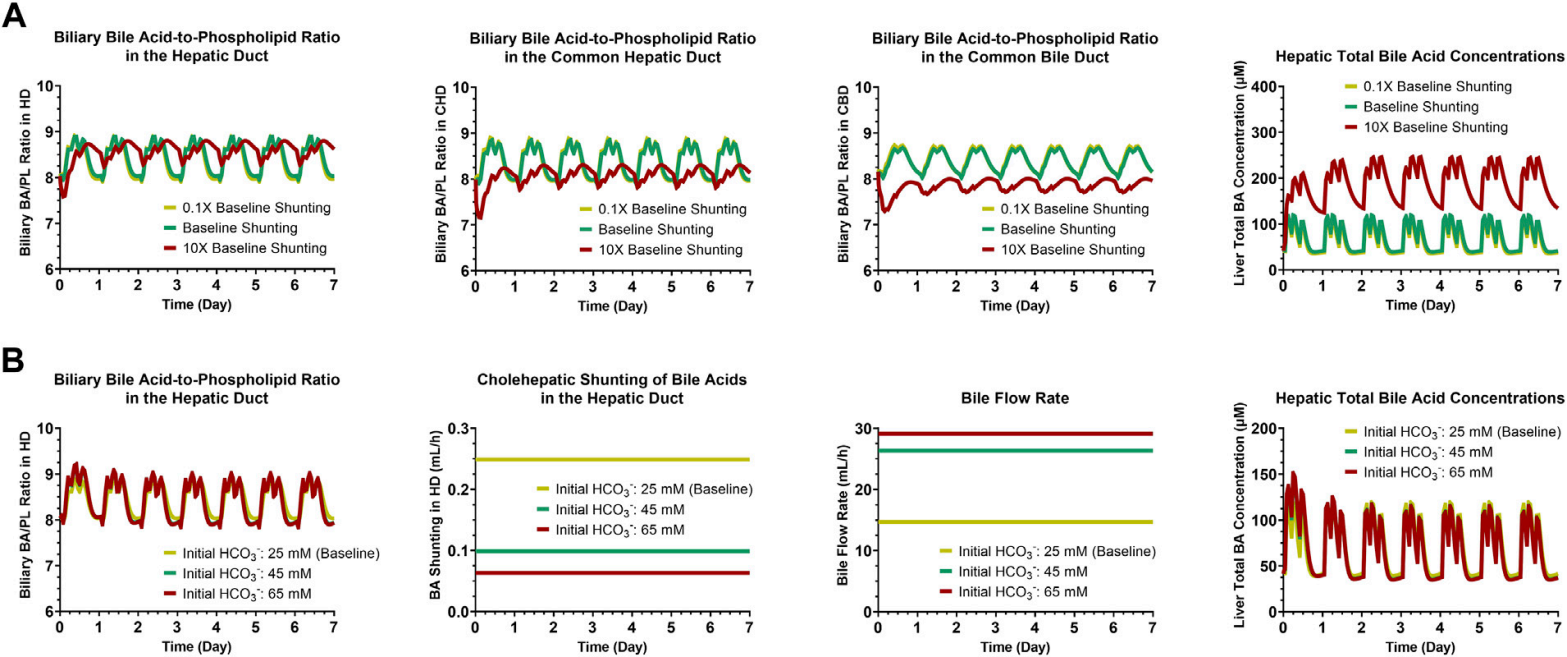
Simulation results for sensitivity analyses of cholehepatic BA shunting **(A)** and initial HCO_3_^−^ concentrations **(B)**. BA, bile acid; CBD, common bile duct; CHD, common hepatic duct; HCO_3_^−^, bicarbonate; HD, hepatic duct; PL, phospholipid.

A sensitivity analysis of cholehepatic BA shunting (0.1X, 1X or 10X the baseline value for each of the three bile duct segments simultaneously: HD, CHD and CBD) demonstrated that an increase in shunting results in a decrease in (the maximum value for) the biliary BA/PL ratio, which became more pronounced for the more downstream bile duct segments (Figure 3A). Although decreasing the BA burden in the bile duct, an increase in cholehepatic BA shunting led to a rise in the concentrations of BAs in hepatocytes. Furthermore, an analysis of increasing the biliary HCO_3_^−^ concentrations from the baseline level (25 mM) to higher concentrations as observed in humans (up to nearly 65 mM) (Matton et al., 2019) showed to have a minor impact on the biliary BA/PL ratio in the HD or on the hepatic BA burden (Figure 3B). However, the increase in biliary HCO_3_^−^ concentrations decreased BA shunting and increased bile flow rate substantially.

**FIGURE 4:**
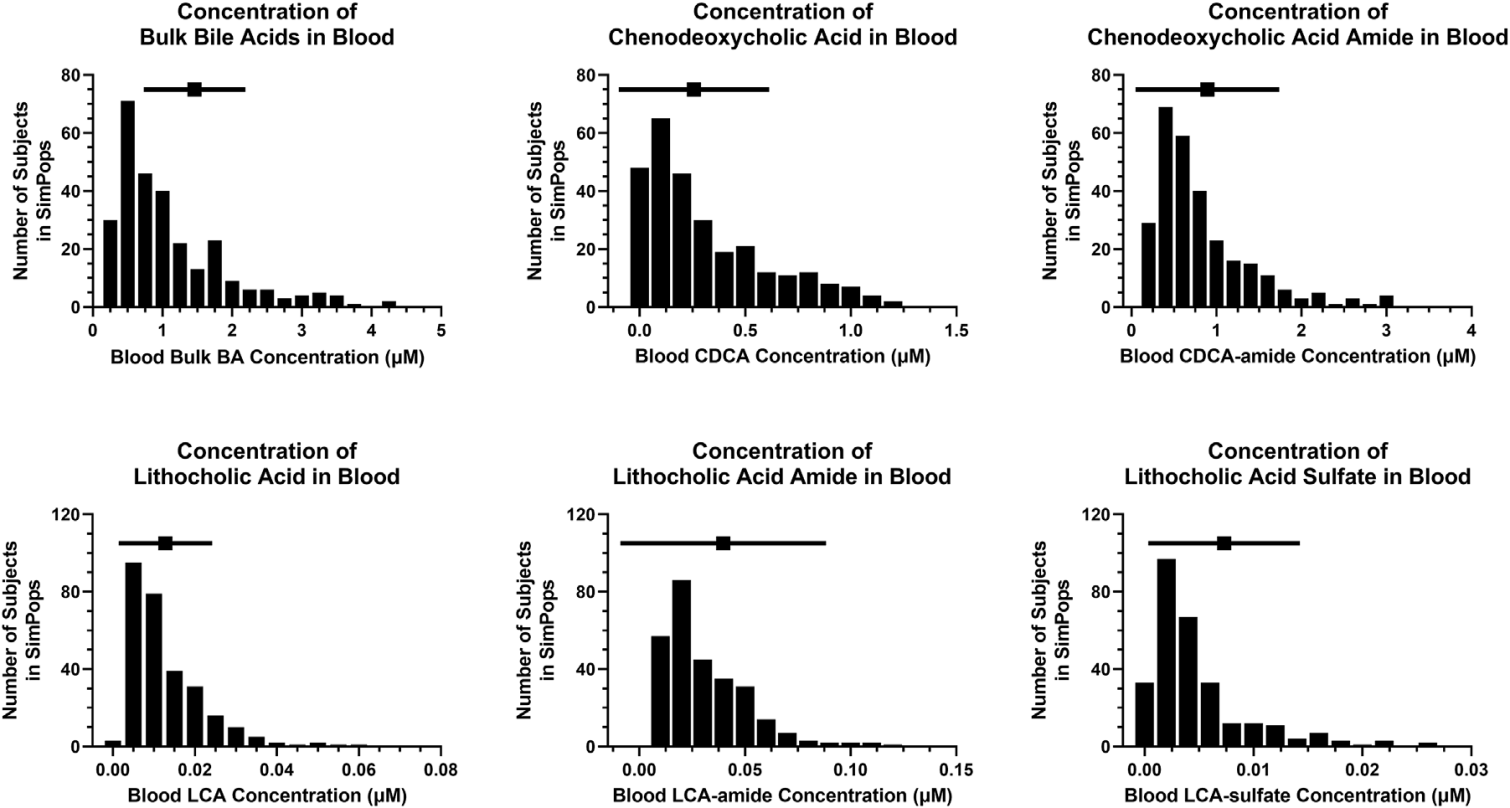
SimPops distribution of blood BA concentrations. BA, bile acid; CDCA, chenodeoxycholic acid; LCA, lithocholic acid. Clinically observed mean ± SD data are depicted by a solid square and horizontal line, respectively; however, circulating BA concentrations were not normally distributed (Trottier et al., 2011).

**FIGURE 5:**
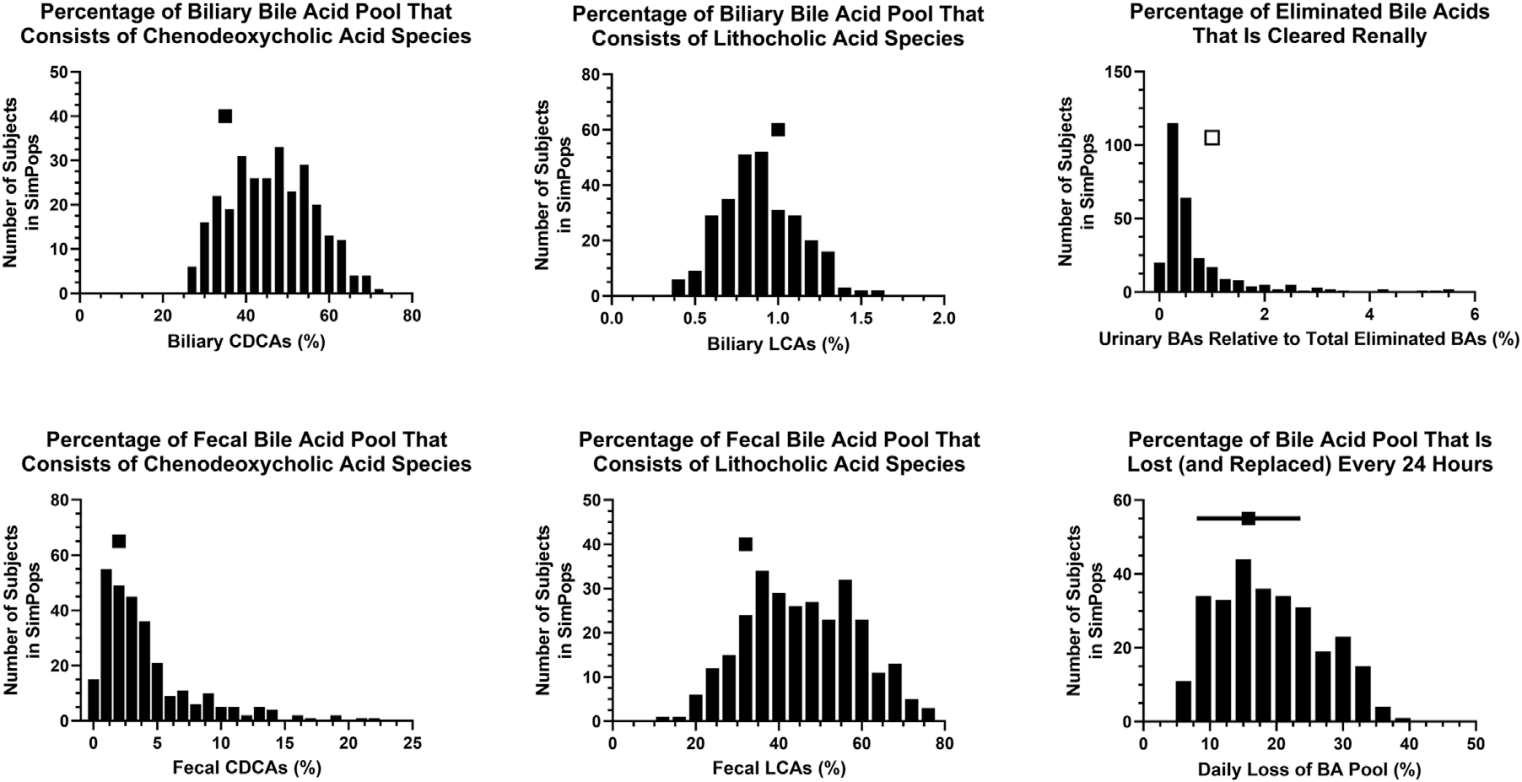
SimPops distribution of BA percentages in different matrices and/or undergoing elimination. BA, bile acid; CDCA, chenodeoxycholic acid; LCA, lithocholic acid. Clinically observed biliary and fecal data are depicted by a single solid square per panel. The percentage of eliminated BAs that is cleared renally has been reported to be <1% (presumably in the majority of individuals), depicted by a single open square. Clinically observed mean ± SD data for the daily loss of the BA pool are depicted by a solid square and horizontal line, respectively.

**FIGURE 6:**
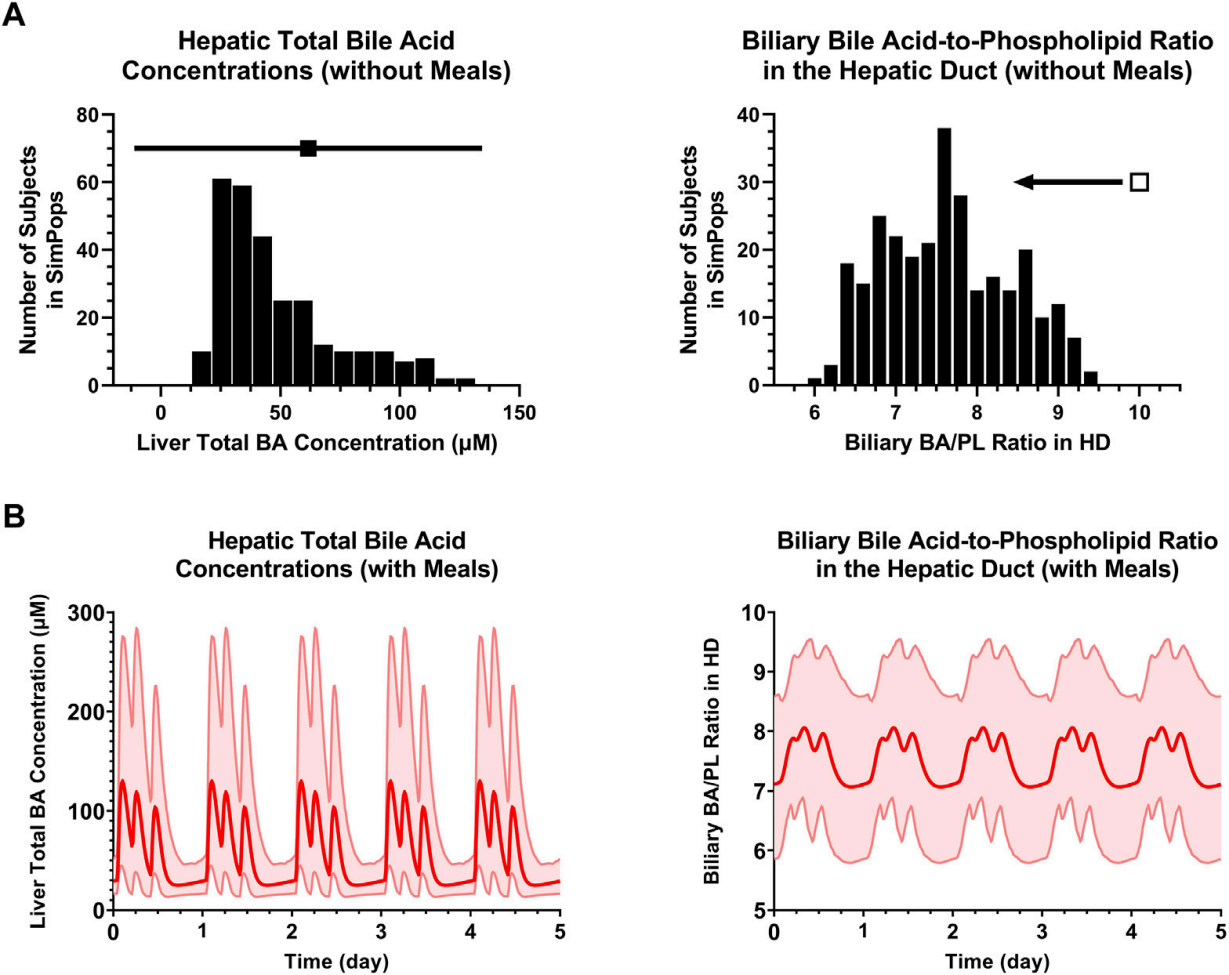
**(A)** SimPops distribution of liver BA concentrations and the biliary BA/PL ratio in the HD under fasted conditions. Clinically observed mean ± SD data for hepatic total BA concentrations (*n* = 6) are depicted by a solid square and horizontal line, respectively. The biliary BA/PL ratio was <10 in the SimPops, as indicated by the single open square and left pointing arrow. **(B)** SimPops average (red profile) and 2.5^th^–97.5^th^ percentiles (light red shading) of liver BA concentrations and the biliary BA/PL ratio in the HD under fed conditions. BA, bile acid; HD, hepatic duct; PL, phospholipid.

A SimPops was developed with the goal to represent population variability in input parameters related to BA and PL homeostasis that can contribute to hepatotoxicity (Table 1), and simultaneously allow model outputs to recapitulate population variability in BA and PL disposition (Figures 4–6), and ultimately DILI outcomes (e.g., plasma ALT > 3x ULN; plasma ALP, GGT). Distributions for blood BA concentrations (Figure 4), biliary and fecal BA composition and BA elimination data (Figure 5) as well as hepatic BA concentrations and the biliary BA/PL ratio (Figure 6A) were informed using the same literature utilized to calibrate the healthy, representative human. As was the case for the representative human, the oscillatory nature of BA disposition due to caloric intake was taken into account when calibrating the human SimPops (Figure 6B). For instance, none of the subjects in the healthy SimPops (*n* = 285) are expected to cross the biliary BA/PL ratio threshold that had been set (i.e., value of 10) that would start eliciting cholestatic liver injury under fasted or fed conditions without drug treatment (Figure 6).

Development of detailed physiologically based pharmacokinetic (PBPK) and *in vitro* toxicity representations for multiple MDR3 inhibitors with known cholestatic liver injury liability is out of scope for the current work. However, the model was further validated at the population level using compounds with known *in vitro* interactions with BA transporters and/or other toxicity mechanisms represented in DILIsym and previously developed PBPK representations. DILI-associated compounds included AMG-009 (Yang et al., 2015; Watkins, 2020), solithromycin (Woodhead et al., 2019) and TAK875 (Longo et al., 2019), while the selection of clean compounds consisted of ambrisentan (Howell et al., 2018), AMG-853 (Watkins, 2020), pioglitazone (Yang K. et al., 2014) and telmisartan (Woodhead et al., 2014b). Since BAs are uncouplers of the mitochondrial H^+^ gradient, the clean drug metformin, which is primarily an inhibitor of the electron transport chain (Yang et al., 2020), was also selected for further validation of the recalibrated BA homeostasis model. SimPops simulations with the new model accurately predicted that ambrisentan (10 mg QD for 12 weeks), AMG-853 (200 mg QD for 12 weeks), pioglitazone (45 mg QD for 2 months), telmisartan (50 mg QD for 30 days) and metformin (1 g QID for 12 weeks) are not associated with hepatocellular nor cholestatic DILI. Furthermore, SimPops simulations using AMG-009 (100 mg BID for 2 weeks; ALT > 3x ULN in 1 out of 8 patients), solithromycin (IV-to-oral dosing: IV 400 mg on days 1–3, PO 800 mg QD on day 4, PO 400 mg QD on days 5–7; ALT > 3x ULN in 5.4%–9.1% of patients) and TAK875 (50 mg QD for 30 days; ALT > 3x ULN in 1.8%–3.2% of type 2 diabetes patients) reasonably recapitulated ALT > 3x ULN: 4.9%, 3.9% and 6.0% using the full SimPops, respectively.

**FIGURE 7:**
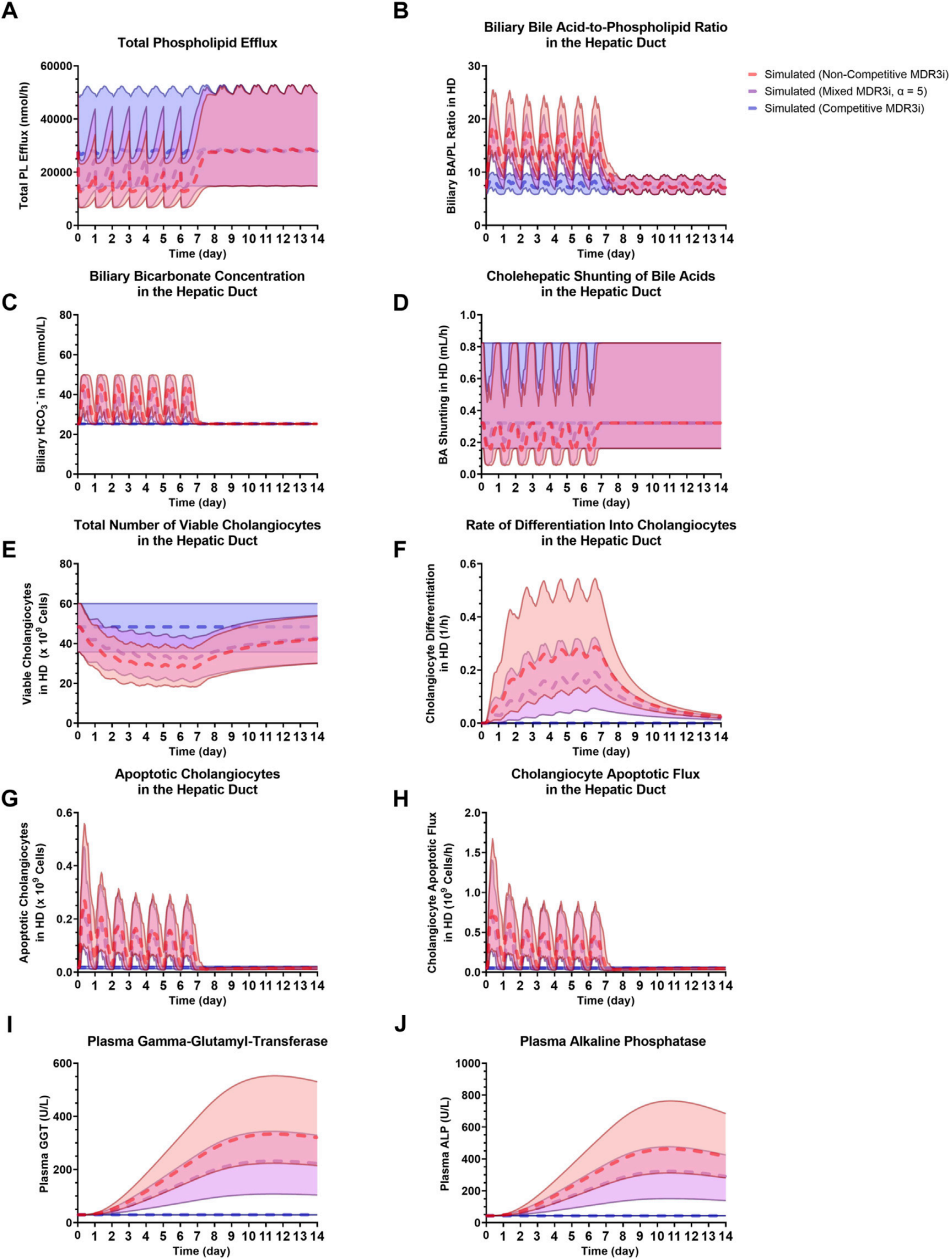
Comparison of non-competitive MDR3 inhibition, mixed MDR3 inhibition (α = 5) and competitive MDR3 inhibition for **(A)** total PL efflux, **(B)** biliary BA/PL ratio in the HD, **(C)** biliary HCO_3_^−^ in the HD, **(D)** BA shunting in the HD, **(E)** viable cholangiocytes in the HD, **(F)** cholangiocyte differentiation in the HD, **(G)** apoptotic cholangiocytes in the HD, **(H)** cholangiocyte apoptotic flux in the HD, **(I)** plasma GGT, **(J)** plasma ALP. Dashed profiles indicate the SimPops average for the three modes of inhibition, while the 2.5^th^–97.5^th^ percentiles of the SimPops are depicted by the transparent shading. ALP, alkaline phosphatase; BA, bile acid; HCO_3_^−^, bicarbonate; HD, hepatic duct; MDR3, multidrug resistance protein 3; MDR3i, MDR3 inhibition; PL, phospholipid.

Using the new cholestatic liver injury submodel and SimPops, a sensitivity analysis of the mode of MDR3 inhibition was performed with an arbitrarily selected 1) compound molecular weight (250 g/mol), 2) MDR3 inhibition coefficient (1 µM), and 3) PK corresponding to QD dosing for 1 week (with simulated C_liver,max,ss_ (mean ± SD) and C_liver,min,ss_ (mean ± SD) = 63.13 ± 14.83 µM and 1.10 ± 0.02 µM, respectively). While competitive inhibition had a negligible impact on PL efflux (Figure 7A) and on downstream, biliary pathways (Figures 7B–J), non-competitive inhibition, and a wide spectrum of mixed inhibition scenarios did predict major disturbances in the bile duct. In the case of BA transporter inhibition, non-competitive and competitive inhibition types have shown to lead to high and low extremes of potential hepatocellular BA accumulation, respectively, whereas mixed inhibition with α = 5 represents a more balanced scenario that has been used when inhibition type is unknown (Generaux et al., 2019; Woodhead et al., 2019). As a representative case for mixed inhibition of MDR3, α = 5 was selected to demonstrate the types of bile duct alterations that were predicted to occur (Figure 7). In summary, the reduction in PL efflux (Figure 7A) resulted in a substantial elevation of the biliary BA/PL ratio in all subjects (Figure 7B), leading to an increase in cholangiocyte apoptosis (Figures 7G, H) and biomarker release (Figures 7I, J). While the number of viable cholangiocytes was simulated to decrease (Figure 7E), differentiation into new cholangiocytes was responsible for cholangiocyte regeneration (Figure 7F). At the same time, the increased biliary BA/PL ratios stimulated the increase in biliary HCO_3_^−^ concentrations (Figure 7C), leading to a temporary reduction in cholehepatic BA shunting (Figure 7D).

## 4 Discussion

In the current work, a QST model of DILI was further developed to include an extended representation of BA homeostasis, in addition to components specifically geared towards investigating clinically defined, cholestatic DILI. Using data from a variety of clinical studies, the model was calibrated and validated under baseline and treatment conditions. Sensitivity analyses showed how the model responds to different modes of MDR3 inhibition, alterations in the rate of cholehepatic BA shunting or biliary concentrations of HCO_3_^−^.

It has been hypothesized that MDR3 inhibition plays a key role in a spectrum of drug-induced cholestatic disorders ranging from bland cholestasis to VBDS (He et al., 2015). Numerous drugs associated with DILI have been found to inhibit MDR3, including itraconazole, ketoconazole, chlorpromazine, imipramine, saquinavir, among others (He et al., 2015; Aleo et al., 2017), some of which are dual MDR3 and BSEP inhibitors, while others primarily inhibit MDR3. The inhibition of MDR3 results in a reduction of biliary availability of PLs for mixed micelle formation with BAs and cholesterol, which can lead to a toxic excess of free BA monomers in the bile that can damage cholangiocytes. Simulations performed in this work have revealed that non-competitive and mixed modes of inhibition are much more likely to increase the biliary BA/PL ratio and lead to cholangiocellular toxicity. While competitive inhibition involves drug and substrate competing for the same binding site on the protein, non-competitive and mixed inhibition involve drug binding to a separate site on the protein, thereby inhibiting its activity. When concentrations of endogenous substrate (e.g., PC) are sufficiently high, a drug that acts as a competitive inhibitor requires to have higher concentrations than a similar non-competitive, or mixed inhibitor to exert an equal inhibitory effect on protein activity. Considering liver concentrations of PC (i.e., 13.3 ± 1.76 nmol/mg tissue), it follows that high concentrations of a competitive MDR3 inhibitor would be required to displace PC from the same binding site, based on the current model. This also explains why the most substantial inhibitory effects on MDR3 are predicted for drugs that act as non-competitive or mixed inhibitors (Figure 7), and why mode of inhibition could be invaluable for hepatotoxicity predictions for an MDR3 inhibitor.

Compared to BA enterohepatic recirculation, cholehepatic BA shunting is an alternative pathway for BAs excreted into bile to return to the sinusoidal blood for hepatocyte uptake. The exact involvement of the cholehepatic shunt pathway in BA disposition is unclear, but is thought to be mostly relevant under cholestatic conditions when biliary BA concentrations are elevated. For instance, apical sodium–BA transporter (Asbt) expressed by rat cholangiocytes has a relatively high K_M_ (Boyer, 2013), suggesting that Asbt-mediated uptake and shunting could be a minor pathway under normal, non-cholestatic conditions. Some types of cholangiocytes have the transporter repertoire (e.g., apical ASBT-mediated uptake followed by basolateral MRP3- and OSTα/β-mediated efflux) that could accommodate vectorial BA transport from bile to sinusoidal blood; however, BA transporter characterization and protein quantification in human primary cholangiocytes is in its infancy and warrants further exploration. Bile duct epithelial cells may show heterogeneous expression of multiple BA transporters, such as in the case of ASBT, which is expressed on the apical membrane in large, but not small cholangiocytes (Boyer, 2013). While cholangiocellular uptake is a concentration-dependent process, an increase in biliary pH reduces cholangiocyte penetration (Hohenester et al., 2012), suggesting that BA-induced HCO_3_^−^ secretion could effectively diminish BA uptake even when BA concentrations are rising, which is accounted for by the model. Although the available literature provides key insights, a detailed quantitative understanding of these interacting factors requires more investigation.

In addition to endogenous BAs and the promising therapeutic 24-norursodeoxycholic acid (norUDCA), which are steroids, the cholehepatic shunt pathway is thought to be utilized by the non-steroidal anti-inflammatory drug sulindac, a known hepatotoxin (Kullak-Ublick, 2013). Similar to norUDCA, sulindac is somewhat resistant to conjugation, and can penetrate cholangiocytes by passive diffusion and induce HCO_3_^−^-rich choleresis (Kullak-Ublick, 2013); furthermore, biliary recovery of sulindac is delayed, consistent with cholehepatic shunting (Bolder et al., 1999). However, when returned to hepatocytes, sulindac is hypothesized to competitively inhibit biliary excretion of BAs resulting in cholestasis, as shown by isolated perfused rat livers co-infused with sulindac and taurocholate (Kullak-Ublick, 2013). These features are concerning for sulindac, but the excellent safety profile for norUDCA suggests that leveraging the cholehepatic shunt pathway could be beneficial depending on the compound. The sensitivity analysis that was performed for the cholehepatic shunt pathway indicated that an increase in shunting (10X of the baseline value) resulted in the hepatic accumulation of BAs (Figure 3A), which includes a component of competitive BA inhibition (in this case by other BA species), similar to sulindac.

The current BA homeostasis model simplifies the representation of glycine- and taurine-conjugated CDCA and LCA species into a category of amidated species (CDCA-amide and LCA-amide), but eventual expansion to an explicit representation of glycine- and taurine-conjugates may be beneficial in relation to the cholehepatic shunt. Per the Henderson-Hasselbalch equation, the pKa of taurine-conjugated CDCA (TCDCA) is approximately 2, while the pKa of CDCA and GCDCA are estimated to exceed a value of 4, indicating that TCDCA protonation is much less amenable to modest physiological changes in biliary pH than CDCA or GCDCA (Hohenester et al., 2011). This hampers TCDCA from being absorbed by cholangiocytes, and indicates that, in terms of cholangiocellular BA accumulation, unconjugated and glycine-conjugated BAs could be considered a more substantial threat (Hohenester et al., 2012). Since the BA pool in rodents is enriched with taurine-conjugated BAs (pKa of 1–2), and is less hepatotoxic in general, this supports why HCO_3_^−^ secretion is more prominent in humans compared to rodents (Hohenester et al., 2012).

The cholestatic liver injury model described herein allows for expansion into other areas related to cholestasis. For instance, mixed micelles extend beyond BAs and PLs, which are the main focus of the currently developed model in order to represent the biliary BA/PL ratio; these mixed micelles are associated with cholesterol as well. Many humans are impacted by biliary cholesterol supersaturation resulting in the formation of cholesterol gallstones (Oude Elferink and Paulusma, 2007). Biliary concentrations of both BAs and PLs contribute to the solubilization of cholesterol. Inhibition of MDR3 results in reduced biliary PLs thereby impairing cholesterol solubilization, which is supported by the observation that Abcb4^−/−^ mice develop cholesterol gallstones (Oude Elferink and Paulusma, 2007). Furthermore, aside from being affected by cholesterol crystals, many patients with cholelithiasis have been found to have MDR3 mutations and a high cholesterol/PL ratio (Rosmorduc et al., 2001).

Other mechanisms of cholestatic DILI that eventually could be incorporated into the model are also recognized, and include direct, toxic effects of drugs and/or metabolites that undergo biliary excretion and interact with cholangiocytes (e.g., α-naphthylisothiocyanate (ANIT), which forms a labile glutathione adduct), or the effects of the immune system (e.g., inflammatory responses) (Padda et al., 2011). In addition to MDR3 inhibition, both of these other cholestatic mechanisms have been linked with the development of VBDS (Padda et al., 2011).

Future applications with the developed model may provide additional insights into its abilities and potential shortcomings that warrant its refinement. Utilization of the model in conjunction with validated PBPK representations of MDR3 inhibitors with and without DILI liability would surely help guide that effort. Also, while new preclinical and clinical data become available, knowledge gaps will be filled, which could justify further model development, recalibration and validation as needed.

In conclusion, the cholestatic liver injury submodel in DILIsym represents several processes important to bile duct viability and toxicity, and shows promise to be useful for predictions of MDR3 inhibition-mediated cholestatic DILI in humans. As the DILIsym platform has shown in past applications (Longo et al., 2016; Woodhead et al., 2018; 2020; 2022; Watkins, 2022), this refined submodel has the potential to inform the clinical development of new drugs, the differentiation of drugs in the same class, and the design of safe drug dosing protocols.

## Data Availability

Upon request, the raw data supporting the conclusions of this article will be made available by the authors, without undue reservation.

## References

[B1] Aleo M. D. Shah F. He K. Bonin P. D. Rodrigues A. D. (2017). Evaluating the role of multidrug resistance protein 3 (MDR3) inhibition in predicting drug-induced liver injury using 125 pharmaceuticals. Chem. Res. Toxicol. 30, 1219–1229. 10.1021/acs.chemrestox.7b00048 28437613

[B2] Andress E. J. Nicolaou M. Romero M. R. Naik S. Dixon P. H. Williamson C. (2014). Molecular mechanistic explanation for the spectrum of cholestatic disease caused by the S320F variant of ABCB4. Hepatology 59, 1921–1931. 10.1002/hep.26970 24806754

[B3] Arab J. P. Cabrera D. Arrese M. (2017). Bile acids in cholestasis and its treatment. Ann. Hepatol. 16, S53–s57. 10.5604/01.3001.0010.5497 31196636

[B4] Baars A. Oosting A. Knol J. Garssen J. van Bergenhenegouwen J. (2015). The gut microbiota as a therapeutic target in IBD and metabolic disease: A role for the bile acid receptors FXR and TGR5. Microorganisms 3, 641–666. 10.3390/microorganisms3040641 27682110 PMC5023267

[B5] Baiocchi L. Zhou T. Liangpunsakul S. Lenci I. Santopaolo F. Meng F. (2019). Dual role of bile acids on the biliary epithelium: Friend or foe? Int. J. Mol. Sci. 20, E1869. 10.3390/ijms20081869 PMC6514722 31014010

[B6] Banales J. M. Huebert R. C. Karlsen T. Strazzabosco M. LaRusso N. F. Gores G. J. (2019). Cholangiocyte pathobiology. Nat. Rev. Gastroenterol. Hepatol. 16, 269–281. 10.1038/s41575-019-0125-y 30850822 PMC6563606

[B7] Beaudoin J. J. Brouwer K. L. R. Malinen M. M. (2020). Novel insights into the organic solute transporter alpha/beta, OSTα/β: From the bench to the bedside. Pharmacol. Ther. 211, 107542. 10.1016/j.pharmthera.2020.107542 32247663 PMC7480074

[B8] Bernhardt G. A. Zollner G. Cerwenka H. Kornprat P. Fickert P. Bacher H. (2012). Hepatobiliary transporter expression and post-operative jaundice in patients undergoing partial hepatectomy. Liver Int. off. J. Int. Assoc. Study Liver 32, 119–127. 10.1111/j.1478-3231.2011.02625.x 22098322

[B9] Bjornsson E. S. Jonasson J. G. (2013). Drug-induced cholestasis. Clin. Liver Dis. 17, 191–209. 10.1016/j.cld.2012.11.002 23540497

[B10] Blidaru D. Blidaru M. Pop C. Crivii C. Seceleanu A. (2010). The common bile duct: Size, course, relations. Rom. J. Morphol. Embryol. 51, 141–144. 20191134

[B11] Bolder U. Trang N. V. Hagey L. R. Schteingart C. D. Ton-Nu H. T. Cerrè C. (1999). Sulindac is excreted into bile by a canalicular bile salt pump and undergoes a cholehepatic circulation in rats. Gastroenterology 117, 962–971. 10.1016/s0016-5085(99)70356-2 10500080

[B12] Bonkovsky H. L. Kleiner D. E. Gu J. Odin J. A. Russo M. W. Navarro V. M. (2017). Clinical presentations and outcomes of bile duct loss caused by drugs and herbal and dietary supplements. Hepatology 65, 1267–1277. 10.1002/hep.28967 27981596 PMC5360519

[B13] Boyer J. L. (2013). Bile formation and secretion. Compr. Physiol. 3, 1035–1078. 10.1002/cphy.c120027 23897680 PMC4091928

[B14] Brevini T. Tysoe O. C. Sampaziotis F. (2020). Tissue engineering of the biliary tract and modelling of cholestatic disorders. J. Hepatol. 73, 918–932. 10.1016/j.jhep.2020.05.049 32535061

[B15] Bruneton J. N. Roux P. Fenart D. Caramella E. Occelli J. P. (1981). Ultrasound evaluation of common bile duct size in normal adult patients and following cholecystectomy. A report of 750 cases. Eur. J. Radiol. 1, 171–172. 7338243

[B16] Buis C. I. Geuken E. Visser D. S. Kuipers F. Haagsma E. B. Verkade H. J. (2009). Altered bile composition after liver transplantation is associated with the development of nonanastomotic biliary strictures. J. Hepatol. 50, 69–79. 10.1016/j.jhep.2008.07.032 19012987

[B17] Chang J. C. Go S. de Waart D. R. Munoz-Garrido P. Beuers U. Paulusma C. C. (2016). Soluble adenylyl cyclase regulates bile salt-induced apoptosis in human cholangiocytes. Hepatology 64, 522–534. 10.1002/hep.28550 26991014 PMC5111777

[B18] Church R. J. Watkins P. B. (2021). The challenge of interpreting alanine aminotransferase elevations in clinical trials of new drug candidates. Clin. Transl. Sci. 14, 434–436. 10.1111/cts.12900 33113257 PMC7993316

[B19] Davit-Spraul A. Gonzales E. Baussan C. Jacquemin E. (2009). Progressive familial intrahepatic cholestasis. Orphanet J. Rare Dis. 4, 1. 10.1186/1750-1172-4-1 19133130 PMC2647530

[B20] Generaux G. Lakhani V. V. Yang Y. Nadanaciva S. Qiu L. Riccardi K. (2019). Quantitative systems toxicology (QST) reproduces species differences in PF-04895162 liver safety due to combined mitochondrial and bile acid toxicity. Pharmacol. Res. Perspect. 7, e00523. 10.1002/prp2.523 31624633 PMC6785660

[B21] Geuken E. Visser D. Kuipers F. Blokzijl H. Leuvenink H. G. D. de Jong K. P. (2004). Rapid increase of bile salt secretion is associated with bile duct injury after human liver transplantation. J. Hepatol. 41, 1017–1025. 10.1016/j.jhep.2004.08.023 15582136

[B22] Gómez Zuleta M. A. Ruíz Morales O. F. Otero Rengino W. A. (2017). What is the normal size of the common bile duct? Rev. Colomb. Gastroenterol. 32, 99. 10.22516/25007440.136

[B23] H. Gray S. Standring H. Ellis B. K. B. Berkovitz (Editors) (2005). Gray’s anatomy: The anatomical basis of clinical practice. 39th ed. (Edinburgh. New York: Elsevier Churchill Livingstone).

[B24] He K. Cai L. Shi Q. Liu H. Woolf T. F. (2015). Inhibition of MDR3 activity in human hepatocytes by drugs associated with liver injury. Chem. Res. Toxicol. 28, 1987–1990. 10.1021/acs.chemrestox.5b00201 26335978

[B25] Higashiyama H. Sumitomo H. Ozawa A. Igarashi H. Tsunekawa N. Kurohmaru M. (2016). Anatomy of the murine hepatobiliary system: A whole-organ-level analysis using a transparency method. Anat. Rec. Hob. 299, 161–172. 10.1002/ar.23287 26559382

[B26] Ho R. H. Leake B. F. Urquhart B. L. Gregor J. C. Dawson P. A. Kim R. B. (2011). Functional characterization of genetic variants in the apical sodium-dependent bile acid transporter (ASBT; SLC10A2). J. Gastroenterol. Hepatol. 26, 1740–1748. 10.1111/j.1440-1746.2011.06805.x 21649730 PMC3170668

[B27] Hoeffel C. Azizi L. Lewin M. Laurent V. Aubé C. Arrivé L. (2006). Normal and pathologic features of the postoperative biliary tract at 3D MR cholangiopancreatography and MR imaging. Radiographics 26, 1603–1620. 10.1148/rg.266055730 17102039

[B28] Hofmann A. F. (1999). The continuing importance of bile acids in liver and intestinal disease. Arch. Intern Med. 159, 2647–2658. 10.1001/archinte.159.22.2647 10597755

[B29] Hohenester S. de Buy Wenniger L. M. Paulusma C. C. van Vliet S. J. Jefferson D. M. Elferink R. P. O. (2012). A biliary HCO3- umbrella constitutes a protective mechanism against bile acid-induced injury in human cholangiocytes. Hepatology 55, 173–183. 10.1002/hep.24691 21932391

[B30] Hohenester S. Maillette de Buy Wenniger L. Jefferson D. M. Oude Elferink R. P. Beuers U. (2011). Biliary bicarbonate secretion constitutes a protective mechanism against bile acid-induced injury in man. Dig. Dis. 29, 62–65. 10.1159/000324687 21691107

[B31] Howell B. A. Ferdous J. Siler S. Q. (2018). Prediction of the liver toxicity of the endothelin receptor antagonists sitaxsentan and ambrisentan for the treatment of pulmonary arterial hypertension with a quantitative systems toxicology tool (DILIsym). Available at: https://www.simulations-plus.com/wp-content/uploads/SOT_2018_Sit_Ambri_DILIsym_FINAL.pdf (Accessed October 14, 2022).

[B32] Howell B. A. Siler S. Q. Barton H. A. Joshi E. M. Cabal A. Eichenbaum G. (2016). Development of quantitative systems pharmacology and toxicology models within consortia: experiences and lessons learned through DILIsym development. Drug Discov. Today Dis. Models 22, 5–13. 10.1016/j.ddmod.2017.04.001

[B33] Howell B. A. Siler S. Q. Watkins P. B. (2014). Use of a systems model of drug-induced liver injury (DILIsym(®)) to elucidate the mechanistic differences between acetaminophen and its less-toxic isomer, AMAP, in mice. Toxicol. Lett. 226, 163–172. 10.1016/j.toxlet.2014.02.007 24560604

[B34] Howell B. A. Yang Y. Kumar R. Woodhead J. L. Harrill A. H. Clewell H. J. 3rd (2012). *In vitro* to *in vivo* extrapolation and species response comparisons for drug-induced liver injury (DILI) using DILIsym^TM^: a mechanistic, mathematical model of DILI. J. Pharmacokinet. Pharmacodyn. 39, 527–541. 10.1007/s10928-012-9266-0 22875368

[B35] Huang L. Zhao A. Lew J.-L. Zhang T. Hrywna Y. Thompson J. R. (2003). Farnesoid X receptor activates transcription of the phospholipid pump MDR3. J. Biol. Chem. 278, 51085–51090. 10.1074/jbc.M308321200 14527955

[B36] Kluth M. Stindt J. Dröge C. Linnemann D. Kubitz R. Schmitt L. (2015). A mutation within the extended X loop abolished substrate-induced ATPase activity of the human liver ATP-binding cassette (ABC) transporter MDR3. J. Biol. Chem. 290, 4896–4907. 10.1074/jbc.M114.588566 25533467 PMC4335229

[B37] Kotronen A. Seppänen-Laakso T. Westerbacka J. Kiviluoto T. Arola J. Ruskeepää A.-L. (2010). Comparison of lipid and fatty acid composition of the liver, subcutaneous and intra-abdominal adipose tissue, and serum. Obes. (Silver Spring) 18, 937–944. 10.1038/oby.2009.326 19798063

[B38] Kullak-Ublick G. A. (2013). Drug-induced cholestatic liver disease. Landes Bioscience. Available at: https://www.ncbi.nlm.nih.gov/books/NBK6102/ (Accessed September 29, 2022).

[B39] Kullak-Ublick G. A. Paumgartner G. Berr F. (1995). Long-term effects of cholecystectomy on bile acid metabolism. Hepatology 21, 41–45. 10.1002/hep.1840210109 7806167

[B40] Lang C. Meier Y. Stieger B. Beuers U. Lang T. Kerb R. (2007). Mutations and polymorphisms in the bile salt export pump and the multidrug resistance protein 3 associated with drug-induced liver injury. Pharmacogenet Genomics 17, 47–60. 10.1097/01.fpc.0000230418.28091.76 17264802

[B41] LaRusso N. F. Korman M. G. Hoffman N. E. Hofmann A. F. (1974). Dynamics of the enterohepatic circulation of bile acids. Postprandial serum concentrations of conjugates of cholic acid in health, cholecystectomized patients, and patients with bile acid malabsorption. N. Engl. J. Med. 291, 689–692. 10.1056/NEJM197410032911401 4851463

[B42] Longo D. M. Woodhead J. L. Walker P. Herédi-Szabó K. Mogyorósi K. Wolenski F. S. (2019). Quantitative systems toxicology analysis of *in vitro* mechanistic assays reveals importance of bile acid accumulation and mitochondrial dysfunction in TAK-875-induced liver injury. Toxicol. Sci. 167, 458–467. 10.1093/toxsci/kfy253 30289550 PMC6358270

[B43] Longo D. M. Yang Y. Watkins P. B. Howell B. A. Siler S. Q. (2016). Elucidating differences in the hepatotoxic potential of tolcapone and entacapone with DILIsym(®), a mechanistic model of drug-induced liver injury. CPT Pharmacometrics Syst. Pharmacol. 5, 31–39. 10.1002/psp4.12053 26844013 PMC4728295

[B44] Ludwig J. Ritman E. L. LaRusso N. F. Sheedy P. F. Zumpe G. (1998). Anatomy of the human biliary system studied by quantitative computer-aided three-dimensional imaging techniques. Hepatology 27, 893–899. 10.1002/hep.510270401 9537426

[B45] Mahdi Z. M. Synal-Hermanns U. Yoker A. Locher K. P. Stieger B. (2016). Role of multidrug resistance protein 3 in antifungal-induced cholestasis. Mol. Pharmacol. 90, 23–34. 10.1124/mol.116.103390 27112167

[B46] Matton A. P. M. de Vries Y. Burlage L. C. van Rijn R. Fujiyoshi M. de Meijer V. E. (2019). Biliary bicarbonate, pH, and glucose are suitable biomarkers of biliary viability during *ex situ* normothermic machine perfusion of human donor livers. Transplantation 103, 1405–1413. 10.1097/TP.0000000000002500 30395120 PMC6613725

[B47] Meier P. J. Stieger B. (2002). Bile salt transporters. Annu. Rev. Physiol. 64, 635–661. 10.1146/annurev.physiol.64.082201.100300 11826283

[B48] Meier Y. Pauli-Magnus C. Zanger U. M. Klein K. Schaeffeler E. Nussler A. K. (2006). Interindividual variability of canalicular ATP-binding-cassette (ABC)-transporter expression in human liver. Hepatology 44, 62–74. 10.1002/hep.21214 16799996

[B49] Mok H. Y. Von Bergmann K. Grundy S. M. (1977). Regulation of pool size of bile acids in man. Gastroenterology 73, 684–690. 10.1016/s0016-5085(19)31766-4 892372

[B50] Morgan R. E. van Staden C. J. Chen Y. Kalyanaraman N. Kalanzi J. Dunn R. T. (2013). A multifactorial approach to hepatobiliary transporter assessment enables improved therapeutic compound development. Toxicol. Sci. official J. Soc. Toxicol. 136, 216–241. 10.1093/toxsci/kft176 23956101

[B51] Morita S. Terada T. (2014). Molecular mechanisms for biliary phospholipid and drug efflux mediated by ABCB4 and bile salts. Biomed. Res. Int. 2014, 954781. 10.1155/2014/954781 25133187 PMC4123595

[B52] Mosedale M. Watkins P. B. (2017). Drug-induced liver injury: Advances in mechanistic understanding that will inform risk management. Clin. Pharmacol. Ther. 101, 469–480. 10.1002/cpt.564 27861792 PMC5359062

[B53] Mosedale M. Watkins P. B. (2020). Understanding idiosyncratic toxicity: Lessons learned from drug-induced liver injury. J. Med. Chem. 63, 6436–6461. 10.1021/acs.jmedchem.9b01297 32037821

[B54] Nakanuma Y. Hoso M. Sanzen T. Sasaki M. (1997). Microstructure and development of the normal and pathologic biliary tract in humans, including blood supply. Microsc. Res. Tech. 38, 552–570. 10.1002/(SICI)1097-0029(19970915)38:6<552::AID-JEMT2>3.0.CO;2-H 9330346

[B55] Napolitano A. Miller S. Nicholls A. W. Baker D. Van Horn S. Thomas E. (2014). Novel gut-based pharmacology of metformin in patients with type 2 diabetes mellitus. PLoS One 9, e100778. 10.1371/journal.pone.0100778 24988476 PMC4079657

[B56] Nosol K. Bang-Sørensen R. Irobalieva R. N. Erramilli S. K. Stieger B. Kossiakoff A. A. (2021). Structures of ABCB4 provide insight into phosphatidylcholine translocation. Proc. Natl. Acad. Sci. U. S. A. 118, e2106702118. 10.1073/pnas.2106702118 34385322 PMC8379956

[B57] Oude Elferink R. P. J. Paulusma C. C. (2007). Function and pathophysiological importance of ABCB4 (MDR3 P-glycoprotein). Pflugers Arch. 453, 601–610. 10.1007/s00424-006-0062-9 16622704

[B58] Padda M. S. Sanchez M. Akhtar A. J. Boyer J. L. (2011). Drug-induced cholestasis. Hepatology 53, 1377–1387. 10.1002/hep.24229 21480339 PMC3089004

[B59] Ramesh Babu C. S. Sharma M. (2014). Biliary tract anatomy and its relationship with venous drainage. J. Clin. Exp. Hepatol. 4, S18–S26. 10.1016/j.jceh.2013.05.002 25755590 PMC4244820

[B60] Rosmorduc O. Hermelin B. Poupon R. (2001). MDR3 gene defect in adults with symptomatic intrahepatic and gallbladder cholesterol cholelithiasis. Gastroenterology 120, 1459–1467. 10.1053/gast.2001.23947 11313316

[B61] Setchell K. D. Rodrigues C. M. Clerici C. Solinas A. Morelli A. Gartung C. (1997). Bile acid concentrations in human and rat liver tissue and in hepatocyte nuclei. Gastroenterology 112, 226–235. 10.1016/s0016-5085(97)70239-7 8978363

[B62] Shoda L. K. Battista C. Siler S. Q. Pisetsky D. S. Watkins P. B. Howell B. A. (2017). Mechanistic modelling of drug-induced liver injury: Investigating the role of innate immune responses. Gene Regul. Syst. Bio 11, 1177625017696074. 10.1177/1177625017696074 PMC5459514 28615926

[B63] Shoda L. K. M. Woodhead J. L. Siler S. Q. Watkins P. B. Howell B. A. (2014). Linking physiology to toxicity using DILIsym(®), a mechanistic mathematical model of drug-induced liver injury. Biopharm. Drug Dispos. 35, 33–49. 10.1002/bdd.1878 24214486

[B64] Stättermayer A. F. Halilbasic E. Wrba F. Ferenci P. Trauner M. (2020). Variants in ABCB4 (MDR3) across the spectrum of cholestatic liver diseases in adults. J. Hepatol. 73, 651–663. 10.1016/j.jhep.2020.04.036 32376413

[B65] Subbiah M. T. Tyler N. E. Buscaglia M. D. Marai L. (1976). Estimation of bile acid excretion in man: comparison of isotopic turnover and fecal excretion methods. J. Lipid Res. 17, 78–84. 10.1016/s0022-2275(20)37020-6 768394

[B66] Tracy T. S. Chaudhry A. S. Prasad B. Thummel K. E. Schuetz E. G. Zhong X.-B. (2016). Interindividual variability in cytochrome P450-mediated drug metabolism. Drug Metab. Dispos. 44, 343–351. 10.1124/dmd.115.067900 26681736 PMC4767386

[B67] Trauner M. Fickert P. Halilbasic E. Moustafa T. (2008). Lessons from the toxic bile concept for the pathogenesis and treatment of cholestatic liver diseases. Wien Med. Wochenschr 158, 542–548. 10.1007/s10354-008-0592-1 18998069

[B68] Trottier J. Białek A. Caron P. Straka R. J. Milkiewicz P. Barbier O. (2011). Profiling circulating and urinary bile acids in patients with biliary obstruction before and after biliary stenting. PLoS ONE 6, e22094. 10.1371/journal.pone.0022094 21760958 PMC3132779

[B69] Turumin J. L. Shanturov V. A. Turumina H. E. (2013). The role of the gallbladder in humans. Rev. Gastroenterol. Mex. 78, 177–187. 10.1016/j.rgmx.2013.02.003 23683886

[B70] Vantrappen G. Rutgeerts P. Ghoos Y. (1981). A new method for the measurement of bile acid turnover and pool size by a double label, single intubation technique. J. Lipid Res. 22, 528–531. 10.1016/s0022-2275(20)34968-3 7017051

[B71] Watkins P. B. (2022). Quantitative systems toxicology and drug development: The DILIsym experience. Methods Mol. Biol. 2486, 181–196. 10.1007/978-1-0716-2265-0_9 35437723

[B72] Watkins P. B. (2020). Quantitative systems toxicology approaches to understand and predict drug-induced liver injury. Clin. Liver Dis. 24, 49–60. 10.1016/j.cld.2019.09.003 31753250

[B73] Woodhead J. L. Howell B. A. Yang Y. Harrill A. H. Clewell H. J. 3rd Andersen M. E. (2012). An analysis of N-acetylcysteine treatment for acetaminophen overdose using a systems model of drug-induced liver injury. J. Pharmacol. Exp. Ther. 342, 529–540. 10.1124/jpet.112.192930 22593093

[B74] Woodhead J. L. Paech F. Maurer M. Engelhardt M. Schmitt-Hoffmann A. H. Spickermann J. (2018). Prediction of safety margin and optimization of dosing protocol for a Novel antibiotic using quantitative systems pharmacology modeling. Clin. Transl. Sci. 11, 498–505. 10.1111/cts.12560 29877622 PMC6132362

[B75] Woodhead J. L. Pellegrini L. Shoda L. K. M. Howell B. A. (2020). Comparison of the hepatotoxic potential of two treatments for autosomal-dominant polycystic kidney DiseaseUsing quantitative systems toxicology modeling. Pharm. Res. 37, 24. 10.1007/s11095-019-2726-0 31909447 PMC6944674

[B76] Woodhead J. L. Siler S. Q. Howell B. A. Watkins P. B. Conway C. (2022). Comparing the liver safety profiles of 4 next-generation CGRP receptor antagonists to the hepatotoxic CGRP inhibitor telcagepant using quantitative systems toxicology modeling. Toxicol. Sci. 188, 108–116. 10.1093/toxsci/kfac051 35556143 PMC9237996

[B77] Woodhead J. L. Yang K. Brouwer K. L. R. Siler S. Q. Stahl S. H. Ambroso J. L. (2014a). Mechanistic modeling reveals the critical knowledge gaps in bile acid-mediated DILI. CPT Pharmacometrics Syst. Pharmacol. 3, e123. 10.1038/psp.2014.21 25006780 PMC4120015

[B78] Woodhead J. L. Yang K. Oldach D. MacLauchlin C. Fernandes P. Watkins P. B. (2019). Analyzing the mechanisms behind macrolide antibiotic-induced liver injury using quantitative systems toxicology modeling. Pharm. Res. 36, 48. 10.1007/s11095-019-2582-y 30734107 PMC6373306

[B79] Woodhead J. L. Yang K. Siler S. Q. Watkins P. B. Brouwer K. L. R. Barton H. A. (2014b). Exploring BSEP inhibition-mediated toxicity with a mechanistic model of drug-induced liver injury. Front. Pharmacol. 5, 240. 10.3389/fphar.2014.00240 25426072 PMC4224072

[B80] Xia X. Francis H. Glaser S. Alpini G. LeSage G. (2006). Bile acid interactions with cholangiocytes. World J. Gastroenterol. 12, 3553–3563. 10.3748/wjg.v12.i22.3553 16773712 PMC4087571

[B81] Yang K. Battista C. Woodhead J. L. Watkins P. B. Howell B. A. Siler S. Q. (2020). Quantitative systems toxicology (QST) modeling suggests that metformin may enhance solithromycin-mediated hepatotoxicity through interactions of toxicological effects, and mitochondrial biogenesis may attenuate hepatotoxicity responses. Available at: https://www.simulations-plus.com/wp-content/uploads/2020-PharmSci-360_poster_K-Yang_QST-Modeling-of-DILI-and-Adaptation.pdf (Accessed October 14, 2022).

[B82] Yang K. Köck K. Sedykh A. Tropsha A. Brouwer K. L. R. (2013). An updated review on drug-induced cholestasis: mechanisms and investigation of physicochemical properties and pharmacokinetic parameters. J. Pharm. Sci. 102, 3037–3057. 10.1002/jps.23584 23653385 PMC4369767

[B83] Yang K. Woodhead J. L. Morgan R. E. Watkins P. B. Howell B. A. Siler S. Q. (2015). Mechanistic modeling with DILIsym® predicts dose-dependent clinical hepatotoxicity of AMG-009 that involves bile acid transporter inhibition. Available at: https://www.simulations-plus.com/wp-content/uploads/Final_AMG009_DILIsym_ACoP_2015.pdf (Accessed October 14, 2022).

[B84] Yang K. Woodhead J. L. Watkins P. B. Howell B. A. Brouwer K. L. R. (2014a). Systems pharmacology modeling predicts delayed presentation and species differences in bile acid-mediated troglitazone hepatotoxicity. Clin. Pharmacol. Ther. 96, 589–598. 10.1038/clpt.2014.158 25068506 PMC4480860

[B85] Yang Y. Nadanaciva S. Will Y. Woodhead J. L. Howell B. A. Watkins P. B. (2014b). MITOsym®: A mechanistic, mathematical model of hepatocellular respiration and bioenergetics. Pharm. Res. 32, 1975–1992. 10.1007/s11095-014-1591-0 25504454 PMC4422870

[B86] Yoshikado T. Takada T. Yamamoto T. Yamaji H. Ito K. Santa T. (2011). Itraconazole-induced cholestasis: involvement of the inhibition of bile canalicular phospholipid translocator MDR3/ABCB4. Mol. Pharmacol. 79, 241–250. 10.1124/mol.110.067256 21056966

