# Supplementary material for: Investigating bile acid-mediated cholestatic drug-induced liver injury using a mechanistic model of multidrug resistance protein 3 (MDR3) inhibition
Source: Front Pharmacol. 2023 Jan 17;13:1085621. doi: 10.3389/fphar.2022.1085621 (PMC9887159; doi:10.3389/fphar.2022.1085621)
Supplement: Supplementary file 1 [file DataSheet1.docx]

**Supplementary Material**

*Bile acid homeostasis*

To briefly summarize key considerations of the bile acid (BA) homeostasis model in DILIsym (**Figure 1A**), BAs are breakdown products of hepatic cholesterol metabolism that undergo conjugation reactions in the liver (e.g., amidation with taurine or glycine, sulfation). While the large majority of BAs are conjugated in the liver prior to undergoing transporter-mediated efflux (e.g., bile salt export pump (BSEP)-mediated biliary excretion, or basolateral multidrug resistance-associated protein (MRP) 3-, MRP4-, organic solute transporter (OST) α/β-mediated efflux), a small fraction of BAs may exit the hepatocyte in unconjugated form as well (e.g., via passive diffusion). Conjugation renders BAs more hydrophilic and less likely to undergo passive diffusion, thus leading to the utilization of transporters for facilitated translocation of conjugated BAs across the plasma membrane. BAs in the sinusoidal blood can enter hepatocytes (e.g., basolateral Na^+^-taurocholate cotransporting polypeptide (NTCP)-mediated uptake) or, when reaching the systemic circulation, be renally eliminated, which is a minor BA elimination pathway. The majority of BAs undergo biliary excretion, pass through the bile duct, and are stored in the gallbladder. Upon meal consumption, meal-related signals lead to the contraction of the gallbladder, thereby releasing stored BAs into downstream portions of the bile duct. Some biliary BAs are believed to penetrate cholangiocytes and be effluxed via the cholangiocellular basolateral membrane to reach the sinusoidal blood and subsequently undergo hepatocellular uptake, constituting the cholehepatic shunt pathway. However, most BAs instead pass through the entire bile duct and are secreted into the duodenal lumen where BAs can undergo gut-mediated metabolism (e.g., deconjugation, dehydroxylation), be absorbed by enterocytes mediated by apical sodium–BA transporter (ASBT) or fecally excreted. When biliary excreted BAs are secreted into the gut, absorbed by intestinal cells to subsequently reach the portal and sinusoidal blood to ultimately enter hepatocytes, a cycle of the enterohepatic circulation (EHC) is assumed to be completed. It is estimated that ~95-99% of the BA pool is maintained during each EHC cycle, while the remaining ~1-5% is eliminated; however, variable numbers of EHC cycles per day have been reported in humans, leading to a larger portion of the BA pool being lost on a daily basis in most individuals (Subbiah et al., 1976; Mok et al., 1977; Vantrappen et al., 1981; Meier and Stieger, 2002). Although dozens of different BA species have been reported in humans, the BA homeostasis model in DILIsym consists of a simplified representation focusing on those most relevant to hepatotoxicity predictions. The hepatically synthesized, primary BA chenodeoxycholic acid (CDCA) is dehydroxylated in the intestinal lumen to form the secondary BA lithocholic acid (LCA), which together constitute a relatively lipophilic BA pair that has been extensively linked to hepatotoxicity. The unconjugated versions of these two BAs as well as amidated CDCA and LCA, and sulfated LCA are explicitly tracked within the BA homeostasis model. All other BA species are lumped together in a “bulk BA” category, which includes the other BA species (e.g., cholic acid (CA), deoxycholic acid (DCA), ursodeoxycholic acid (UDCA)). BAs are ligands for the hepatic nuclear farnesoid X receptor (FXR), which is an important regulator of BA homeostasis. When BAs accumulate in the hepatocyte (e.g., due to drug-induced inhibition of BA efflux transporters), FXR is activated and regulates several hepatoprotective pathways, including the downregulation of BA synthesis and BA uptake transporter levels, and the upregulation of basolateral and canalicular BA efflux transporters, which collectively decrease the hepatic BA burden. Because *in vitro* experiments suggest that CDCA species are more potent FXR agonists than other BA species (Jonker et al., 2012), the model triggers FXR-based adaptation in response to hepatic CDCA and CDCA-amide levels. When adaptation pathways are not able to maintain hepatocellular BA levels at sufficiently low levels, accumulation of BAs will disrupt cellular energy balance in the model (e.g., by uncoupling the mitochondrial H^+^ gradient), thereby impacting the hepatocyte life cycle, and releasing drug-induced liver injury (DILI) biomarkers associated with hepatocellular injury (e.g., alanine and aspartate aminotransferases, ALT and AST) or overall liver dysfunction (e.g., total bilirubin), all of which are represented in the model and supported by the literature.

*Biliary bile acid-to-phospholipid ratio*

The biliary BA/phospholipid (PL) ratio can be used to represent mixed micelle formation, and in the case of a high ratio, reflects a toxic surplus of free BA monomers in the bile. The BA/PL ratio has been associated with cholestatic liver injury in several studies (Geuken et al., 2004; Buis et al., 2009; Davit-Spraul et al., 2009). In the context of nonanastomotic strictures (NAS) of the bile duct, which is considered a troublesome post-transplant cholangiopathy involving strictures of the intrahepatic and mainly extrahepatic bile ducts (de Vries et al., 2018), relatively high BA/PL ratios were associated with worse biliary outcomes (e.g., higher bile duct injury scores) compared to lower BA/PL ratios (Geuken et al., 2004; Buis et al., 2009). It has been hypothesized that BA toxicity, presumably attributable to free BA monomers, contributes to the poorly understood pathogenesis of NAS. Additionally, in patients with progressive familial cholestasis (PFIC) type 3, the biliary BA/PL ratio is approximately 5-fold higher compared to healthy controls. The specific *ABCB4* mutations in individual patients with PFIC3 seem to dictate the residual biliary PL levels (Davit-Spraul et al., 2009). PFIC3 patients with a higher concentration of remaining biliary PLs (>7% of total biliary lipids), and thus a relatively lower BA/PL ratio, appear to respond better to UDCA therapy (Davit-Spraul et al., 2009), which is a promising treatment for some cholestatic disorders, having shown clinically beneficial effects in primary biliary cholangitis (PBC), but unfortunately not in primary sclerosing cholangitis (PSC) (Ali et al., 2017).

*Cholehepatic shunting of bile acids*

Cholehepatic shunting likely involves both passive diffusion and cholangiocellular transport processes. Investigations in rats suggest that BA transporters that play a role in BA transport in hepatocytes and/or intestinal cells are also expressed in cholangiocytes (Benedetti et al., 1997; Kagawa, 2017; Banales et al., 2019). These include ASBT on the apical membrane, and MRP3, OSTα/β and a truncated form of ASBT (t-ASBT) on the basolateral membrane, providing the transporter repertoire needed for vectorial transport of conjugated BAs across the cholangiocyte plasma membrane from bile to the blood of the peribiliary plexus (Xia et al., 2006; Arab et al., 2017). At least some of these findings in rodents may be translatable to humans, since MRP3, which has wide BA substrate selectivity, is highly expressed on the basolateral membrane of human cholangiocytes (Hirohashi et al., 2000). Furthermore, patients with various forms of cholestasis demonstrated MRP3 protein increases in cholangiocytes, supporting a protective role for cholehepatic shunting of BAs (Scheffer et al., 2002). Protonated and unconjugated BAs can undergo passive cholangiocellular absorption (Arab et al., 2017; Banales et al., 2019), but the large majority of endogenous, hepatocellular BAs excreted into bile are conjugated, which are less likely to undergo passive diffusion. A promising therapeutic, 24-norursodeoxycholic acid (norUDCA), currently in clinical development for PSC patients is hypothesized to utilize the cholehepatic shunt pathway. NorUDCA is a sidechain-shortened version of the relatively hydrophilic, naturally occurring UDCA, and is largely resistant to amidation (Samant et al., 2019), which promotes its protonation and thereby its penetration into cholangiocytes (Halilbasic et al., 2017). Cholehepatic shunting of norUDCA induces bicarbonate (HCO_3_^-^)-rich hypercholeresis, which protects against biliary BA toxicity (Samant et al., 2019). UDCA therapy also leads to HCO_3_^-^ secretion, but via a mechanism different from cholehepatic shunting, and is furthermore believed to decrease the lipophilicity of the BA pool by replacing toxic BAs with UDCA, a relatively non-toxic BA species (Padda et al., 2011; Halilbasic et al., 2017). While the safety profile of norUDCA is excellent and significantly reduced alkaline phosphatase (ALP) levels in a dose-dependent manner during a 12-week treatment of PSC patients (Fickert et al., 2017), UDCA has shown to be toxic at higher doses presumably due to increased biliary BA concentrations (Halilbasic et al., 2017).

*Bicarbonate secretion*

A glycocalyx, a 20-30-nm-thick layer on the apical membrane of cholangiocytes as identified by electron microscopy, is believed to stabilize the alkaline pH microclimate in the bile duct and protect cholangiocytes against free biliary BAs, giving rise to the so-called “bicarbonate umbrella” (Hohenester et al., 2012; Arab et al., 2017). Destabilization of this glycocalyx via desialylation (e.g., by neuraminidase treatment) increased susceptibility to BA-induced toxicity in a pH-dependent manner (Hohenester et al., 2012). The HCO_3_^-^ umbrella is hypothesized to enable deprotonation of BAs in close proximity to the apical cholangiocellular plasma membrane (Hohenester et al., 2012). Interestingly, the gene encoding for the fucosyltransferase 2 enzyme involved in glycocalyx maintenance was found to be a risk factor for PSC (Hohenester et al., 2012). Furthermore, AE2 expression is downregulated in PBC, resulting in impaired HCO_3_^-^ secretion (Banales et al., 2019).

The secretion of biliary HCO_3_^-^ is triggered by the gastrointestinal hormone secretin during the postprandial period (Banales et al., 2019), resulting in a signaling cascade ultimately leading to AE2-mediated efflux of HCO_3_^-^. Secretin-stimulated biliary HCO_3_^-^ secretion is dependent on BA concentrations (Banales et al., 2019), which is supported by reports that biliary BA concentrations and composition regulate secretory function of cholangiocytes (Alpini et al., 1999; Arab et al., 2017). The G protein-coupled BA receptor 1 (GPBAR1), or TGR5, resides on the tip of the cholangiocellular cilia in intra- and extrahepatic bile ducts that reach beyond the HCO_3_^-^ umbrella, and is able to detect biliary BAs (Beuers et al., 2010). Data support a role for biliary BAs mediating a TGR5-based signaling cascade leading to biliary HCO_3_^-^ secretion (Beuers et al., 2010; Boyer, 2013; Duboc et al., 2014). The gene encoding for TGR5 has been found to be a risk factor in PSC, potentially impacting the function of the biliary HCO_3_^-^ umbrella (Beuers et al., 2010). TGR5 can be activated by a wide range of unconjugated and conjugated BAs (Duboc et al., 2014; Fiorucci and Distrutti, 2019). Experiments measuring downstream TGR5 signaling (i.e., cAMP production) suggest that TGR5 is activated in a dose-dependent manner by BAs in the following rank order: LCA≥DCA>CDCA>CA, indicating that TGR5 activation is most sensitive to the relatively lipophilic and hepatotoxic BA species (Duboc et al., 2014). LCA is considered the most potent endogenous agonist for TGR5, with an EC_50_ of 530 nM (Thomas et al., 2008).

**References**

Ali, A. H., Tabibian, J. H., and Lindor, K. D. (2017). Update on pharmacotherapies for cholestatic liver disease. *Hepatol Commun* 1, 7–17. doi: 10.1002/hep4.1013.

Alpini, G., Glaser, S. S., Ueno, Y., Rodgers, R., Phinizy, J. L., Francis, H., et al. (1999). Bile acid feeding induces cholangiocyte proliferation and secretion: evidence for bile acid-regulated ductal secretion. *Gastroenterology* 116, 179–186. doi: 10.1016/s0016-5085(99)70242-8.

Arab, J. P., Cabrera, D., and Arrese, M. (2017). Bile Acids in Cholestasis and its Treatment. *Ann Hepatol* 16, s53–s57. doi: 10.5604/01.3001.0010.5497.

Banales, J. M., Huebert, R. C., Karlsen, T., Strazzabosco, M., LaRusso, N. F., and Gores, G. J. (2019). Cholangiocyte pathobiology. *Nat Rev Gastroenterol Hepatol* 16, 269–281. doi: 10.1038/s41575-019-0125-y.

Benedetti, A., Di Sario, A., Marucci, L., Svegliati-Baroni, G., Schteingart, C. D., Ton-Nu, H. T., et al. (1997). Carrier-mediated transport of conjugated bile acids across the basolateral membrane of biliary epithelial cells. *Am J Physiol* 272, G1416-1424. doi: 10.1152/ajpgi.1997.272.6.G1416.

Beuers, U., Hohenester, S., de Buy Wenniger, L. J. M., Kremer, A. E., Jansen, P. L. M., and Elferink, R. P. J. O. (2010). The biliary HCO(3)(-) umbrella: a unifying hypothesis on pathogenetic and therapeutic aspects of fibrosing cholangiopathies. *Hepatology* 52, 1489–1496. doi: 10.1002/hep.23810.

Boyer, J. L. (2013). Bile formation and secretion. *Compr Physiol* 3, 1035–1078. doi: 10.1002/cphy.c120027.

Buis, C. I., Geuken, E., Visser, D. S., Kuipers, F., Haagsma, E. B., Verkade, H. J., et al. (2009). Altered bile composition after liver transplantation is associated with the development of nonanastomotic biliary strictures. *J Hepatol* 50, 69–79. doi: 10.1016/j.jhep.2008.07.032.

Davit-Spraul, A., Gonzales, E., Baussan, C., and Jacquemin, E. (2009). Progressive familial intrahepatic cholestasis. *Orphanet J Rare Dis* 4, 1. doi: 10.1186/1750-1172-4-1.

de Vries, Y., von Meijenfeldt, F. A., and Porte, R. J. (2018). Post-transplant cholangiopathy: Classification, pathogenesis, and preventive strategies. *Biochim Biophys Acta Mol Basis Dis* 1864, 1507–1515. doi: 10.1016/j.bbadis.2017.06.013.

Duboc, H., Taché, Y., and Hofmann, A. F. (2014). The bile acid TGR5 membrane receptor: from basic research to clinical application. *Dig Liver Dis* 46, 302–312. doi: 10.1016/j.dld.2013.10.021.

Fickert, P., Hirschfield, G. M., Denk, G., Marschall, H.-U., Altorjay, I., Färkkilä, M., et al. (2017). norUrsodeoxycholic acid improves cholestasis in primary sclerosing cholangitis. *J Hepatol* 67, 549–558. doi: 10.1016/j.jhep.2017.05.009.

Fiorucci, S., and Distrutti, E. eds. (2019). *Bile Acids and Their Receptors*. Cham: Springer International Publishing doi: 10.1007/978-3-030-22005-1.

Geuken, E., Visser, D., Kuipers, F., Blokzijl, H., Leuvenink, H. G. D., de Jong, K. P., et al. (2004). Rapid increase of bile salt secretion is associated with bile duct injury after human liver transplantation. *J Hepatol* 41, 1017–1025. doi: 10.1016/j.jhep.2004.08.023.

Halilbasic, E., Steinacher, D., and Trauner, M. (2017). Nor-Ursodeoxycholic Acid as a Novel Therapeutic Approach for Cholestatic and Metabolic Liver Diseases. *Dig Dis* 35, 288–292. doi: 10.1159/000454904.

Hirohashi, T., Suzuki, H., Takikawa, H., and Sugiyama, Y. (2000). ATP-dependent transport of bile salts by rat multidrug resistance-associated protein 3 (Mrp3). *J Biol Chem* 275, 2905–2910. doi: 10.1074/jbc.275.4.2905.

Hohenester, S., Wenniger, L. M. de B., Paulusma, C. C., van Vliet, S. J., Jefferson, D. M., Elferink, R. P. O., et al. (2012). A biliary HCO3- umbrella constitutes a protective mechanism against bile acid-induced injury in human cholangiocytes. *Hepatology* 55, 173–183. doi: 10.1002/hep.24691.

Jonker, J. W., Liddle, C., and Downes, M. (2012). FXR and PXR: potential therapeutic targets in cholestasis. *J Steroid Biochem Mol Biol* 130, 147–158. doi: 10.1016/j.jsbmb.2011.06.012.

Kagawa, T. (2017). “Hepatobiliary Transport of Bile Acids,” in *Bile Acids in Gastroenterology*, eds. S. Tazuma and H. Takikawa (Tokyo: Springer Japan), 9–25. doi: 10.1007/978-4-431-56062-3_2.

Meier, P. J., and Stieger, B. (2002). Bile salt transporters. *Annu Rev Physiol* 64, 635–661. doi: 10.1146/annurev.physiol.64.082201.100300.

Mok, H. Y., Von Bergmann, K., and Grundy, S. M. (1977). Regulation of pool size of bile acids in man. *Gastroenterology* 73, 684–690.

Padda, M. S., Sanchez, M., Akhtar, A. J., and Boyer, J. L. (2011). Drug-induced cholestasis. *Hepatology* 53, 1377–1387. doi: 10.1002/hep.24229.

Samant, H., Manatsathit, W., Dies, D., Shokouh-Amiri, H., Zibari, G., Boktor, M., et al. (2019). Cholestatic liver diseases: An era of emerging therapies. *World J Clin Cases* 7, 1571–1581. doi: 10.12998/wjcc.v7.i13.1571.

Scheffer, G. L., Kool, M., de Haas, M., de Vree, J. M. L., Pijnenborg, A. C. L. M., Bosman, D. K., et al. (2002). Tissue distribution and induction of human multidrug resistant protein 3. *Lab. Invest.* 82, 193–201.

Subbiah, M. T., Tyler, N. E., Buscaglia, M. D., and Marai, L. (1976). Estimation of bile acid excretion in man: comparison of isotopic turnover and fecal excretion methods. *J Lipid Res* 17, 78–84.

Thomas, C., Pellicciari, R., Pruzanski, M., Auwerx, J., and Schoonjans, K. (2008). Targeting bile-acid signalling for metabolic diseases. *Nat Rev Drug Discov* 7, 678–693. doi: 10.1038/nrd2619.

Vantrappen, G., Rutgeerts, P., and Ghoos, Y. (1981). A new method for the measurement of bile acid turnover and pool size by a double label, single intubation technique. *J Lipid Res* 22, 528–531.

Xia, X., Francis, H., Glaser, S., Alpini, G., and LeSage, G. (2006). Bile acid interactions with cholangiocytes. *World J Gastroenterol* 12, 3553–3563. doi: 10.3748/wjg.v12.i22.3553.
